# High-speed photonic neuromorphic computing using recurrent optical spectrum slicing neural networks

**DOI:** 10.1038/s44172-022-00024-5

**Published:** 2022-10-26

**Authors:** Kostas Sozos, Adonis Bogris, Peter Bienstman, George Sarantoglou, Stavros Deligiannidis, Charis Mesaritakis

**Affiliations:** 1 Dept. of Informatics and Computer Engineering, University of West Attica, Egaleo, Greece; 2 Dept. of Information Technology, Ghent University-imec, Gent, Belgium; 3 Dept. Information and Communication Systems Engineering, Engineering School, University of the Aegean, Samos, Greece

**Keywords:** Computer science, Electrical and electronic engineering, Photonic devices

## Abstract

Neuromorphic computing using photonic hardware is a promising route towards ultrafast processing while maintaining low power consumption. Here we present and numerically evaluate a hardware concept for realizing photonic recurrent neural networks and reservoir computing architectures. Our method, called Recurrent Optical Spectrum Slicing Neural Networks (ROSS-NNs), uses simple optical filters placed in a loop, where each filter processes a specific spectral slice of the incoming optical signal. The synaptic weights in our scheme are equivalent to the filters’ central frequencies and bandwidths. Numerical application to high baud rate optical signal equalization (>100 Gbaud) reveals that ROSS-NN extends optical signal transmission reach to > 60 km, more than four times that of two state-of-the-art digital equalizers. Furthermore, ROSS-NN relaxes complexity, requiring less than 100 multiplications/bit in the digital domain, offering tenfold reduction in power consumption with respect to these digital counterparts. ROSS-NNs hold promise for efficient photonic hardware accelerators tailored for processing high-bandwidth (>100 GHz) optical signals in optical communication and high-speed imaging applications.

## Abstract (web summary)

Sozos and co-workers present and numerically evaluate photonic neuromorphic hardware using recurrent optical spectrum slicing for use in ultra-fast optical applications. The approach extends optical signal transmission reach to more than four-fold that of two state-of-the-art digital equalizers and reduces power consumption tenfold.

## Introduction

Recurrent Neural Networks (RNNs) are universal computational tools tailored to process time-dependent data^1^. State-of-the-Art RNN architectures, such as Long Short-Term Memory, Bi-directional RNNs or Gated-Recurrent-Units^2,3^ remain notoriously difficult to train, requiring the optimization of a significant number of hyper-parameters. Furthermore, the practicality of RNNs becomes even more questionable, when multi-GHz data inference is required by demanding applications in the area of optical communications and imaging. Up to now, RNN’s superiority over other nonlinear digital signal processing techniques has been proved only through offline signal processing. Unfortunately, their realization by field programmable gate arrays or application-specific integrated circuits constitutes a quite challenging task, especially if processing rates exceeding 50 Gbaud are targeted^4^. Aiming to amend these drawbacks, Reservoir Computing (RC) has emerged as a neuromorphic paradigm that offers radical simplification of the cumbersome RNN training^5^. In detail, by splitting the recurrent network in a reservoir (hidden layer) with random and untrained weights and a readout layer, where all training is taking place in a linear manner, RC reduces complexity, while retaining performance. Moreover, from a hardware perspective, the randomness of the reservoir layer does not translate to performance deterioration, but on the contrary provides robustness against fabrication imperfections. These unique features of RCs render them a hardware-friendly solution for various implementations, exploiting diverse platforms ranging from spintronics^6^, polaritons, CMOS electronics^7^ to free-space optics^8,9^ and integrated photonic-based approaches^10^. Especially photonics technology constitutes a proliferating platform for such schemes, due to inherent advantages such as computational parallelism through signal multiplexing, low power consumption, high-bandwidth support and processing at the speed of light^10^. These merits are exploited to the maximum in applications where the information to be processed is already in the optical domain, therefore direct complex processing can be obtained, alleviating the need for power-hungry optoelectronic and electro-optical conversions. On the other hand, although photonics is suitable for implementing linear transformations using passive components^11,12^, it fails to provide integrated and low-power non-linear nodes, which is a critical part of an RC/RNN architecture.

In a photonic RC context, most efforts have concentrated on the rich non-linear dynamics of semiconductor lasers subjected to feedback. These schemes, when combined with time-multiplexing, have proved their efficacy in addressing difficult problems like time-series prediction^13–15^, image recognition^16^, non-linear channel equalization^17,18^ or chromatic dispersion (CD) compensation in intensity modulation/direct detection (IM/DD) transmission systems^19,20^. This sub-category of photonic RC, called time-delayed RC in the literature, has minimum photonic hardware requirements, consisting of a single nonlinear physical node and multiple time-multiplexed virtual nodes. Nevertheless, it is not compatible with all-optical coherent processing and is not integration friendly due to the fact that the number of nodes is proportional to the length of the external delay path. For the same reason, time-delayed systems may achieve real-time signal processing only up to 20 Gbaud as, the smaller the symbol period, the lower the number of virtual nodes that can be exploited for processing, thus affecting processing power. To make matters worse, in time-delayed RCs, a high-speed pseudo-random generator is also needed so as to mask the incoming signal, thus evoking differentiation between the dynamics of the virtual nodes. This unavoidable requirement hinders all-optical implementations and increases the digital processing requirements. A different implementation strategy consists of RC or RNN with spatially distributed nodes that usually contain passive waveguides^21^, spatial sampling positions across the complex multimode field of an injection-locked vertical cavity surface emitting laser^22^, semiconductor optical amplifiers^23^ or micro-ring resonators (MRRs)^24,25^. In terms of a node’s non-linearity, a limited number of solutions have been proposed, either demanding power-hungry active elements, power-demanding nonlinear phase shifters relying on Kerr effect or are based on the square law offered by the photodiodes at the output layer. The use of photonic components for realizing RC nodes offers practically unlimited processing speed without sacrificing coherent processing. This feature outweighs all spatial RC’s restrictions when high-speed applications are considered. In this context, photonic RC has attracted attention in the optical communications field, thanks to its ability to compensate transmission impairments such as CD-induced power fading and Kerr-related non-linearities^26^. All the aforementioned works do not rely on any type of spectral slicing and optical processing of selected frequency components in the optical domain.

In this work, we numerically explore a recurrent photonic integrated node consisting of a hardware-friendly filter-in-a-loop architecture that harnesses computational efficiency in a two-fold manner. First, the proposed architecture implements spectral slicing of the incoming signal through a complex node response, directly in the optical domain, leading to spectral decomposition of the signal, which is a prerequisite when broadband optical signals (from 100 GHz to several THz) are to be processed. Spectral slices consist of lower-bandwidth components of the original signal offering the possibility of diverse and specialized treatment of information in the frequency domain. By arbitrarily manipulating the frequency/phase information of the signal, random weighting is applied in the spectral domain. Second, although an optical filter performs a linear transformation of the complex field, it also provides a non-linear mapping of the incoming signal’s phase variations to the node’s intensity (see Supplementary Discussion 1)^27^. Hence, the coherent interaction of spectrally sliced components on the photodiode provides a complex nonlinear activation function at the output. This filter-based neuromorphic assumption of nodes is inspired by the filter-and-fire model^28^ which treats each retinal ganglion cell as a linear filter followed by a nonlinear activation function. Spectral slicing, through simple non-recurrent filters, before RC processing was first proposed in^29,30^ where the output of each filter was sent to a digitally implemented reservoir computing network. Nonetheless, this approach is not advantageous in terms of power consumption because the recurrent processing still lies in the digital domain. In such a case, the digital complexity remains high, important phase information is not provided to the processing system and the spectral processing is incomplete and limited. Here, we implement a fully photonic structure based on filters-in-a-loop. Based on the functionality of the photonic node, we call the proposed architecture Recurrent Optical Spectrum Slicing Neural Network (ROSS-NN). Each node of the ROSS-NN exhibits truly passive operation, and there is no intrinsic bandwidth limitation burdening the proposed scheme. Processing speed is capped only by the bandwidth of the photodiodes and the analog-to-digital conversion. A ROSS-NN can be incorporated either as an RNN or spatial RC architecture, providing direct coherent processing without costly electro-optic conversions, speed processing penalty, high-speed pseudo-random generators for mask realization^13^ and most importantly, with marginal power consumption. In the RC case, the application of weights is implemented at a digital output layer. Thus, through parallelization, the weighting of the RC outputs can be performed with a speed, matching the digitization process. Aiming to demonstrate the merits of ROSS-NN, we have numerically investigated its processing capabilities in two tasks. First, we confirm its nonlinear processing nature by exhibiting its efficacy in generic non-linear tasks, such as inferring the behavior of unseen data of a dynamical system using multi-Gbaud rates. Second, and more importantly, we also numerically demonstrate its performance in real-life problems such as the mitigation of transmission impairments caused by CD and Kerr effect in IM/DD systems at 112 Gbaud PAM-4 as well as in coherent systems employing QAM-16 signals. ROSS-NN exhibits improved performance over well-established techniques such as Maximum Likelihood Sequence Estimation (MLSE) and Volterra Non-Linear Equalizers (VNLE) and other photonic neuromorphic approaches, extending the reach of high-speed IM/DD systems far beyond the 10–15 km limit of the digital algorithms. The bit-error-rate (BER) achieved by ROSS-NN is only limited by noise, as in typical linear channels. The simulated system achieves real-time processing with almost zero latency at tasks exceeding 100 Gbaud. In this work, numerical simulations show that ROSS-NN can operate at timescales of a few picoseconds.

## Results

### ROSS-NN node and overall architecture

**Fig. 1:**
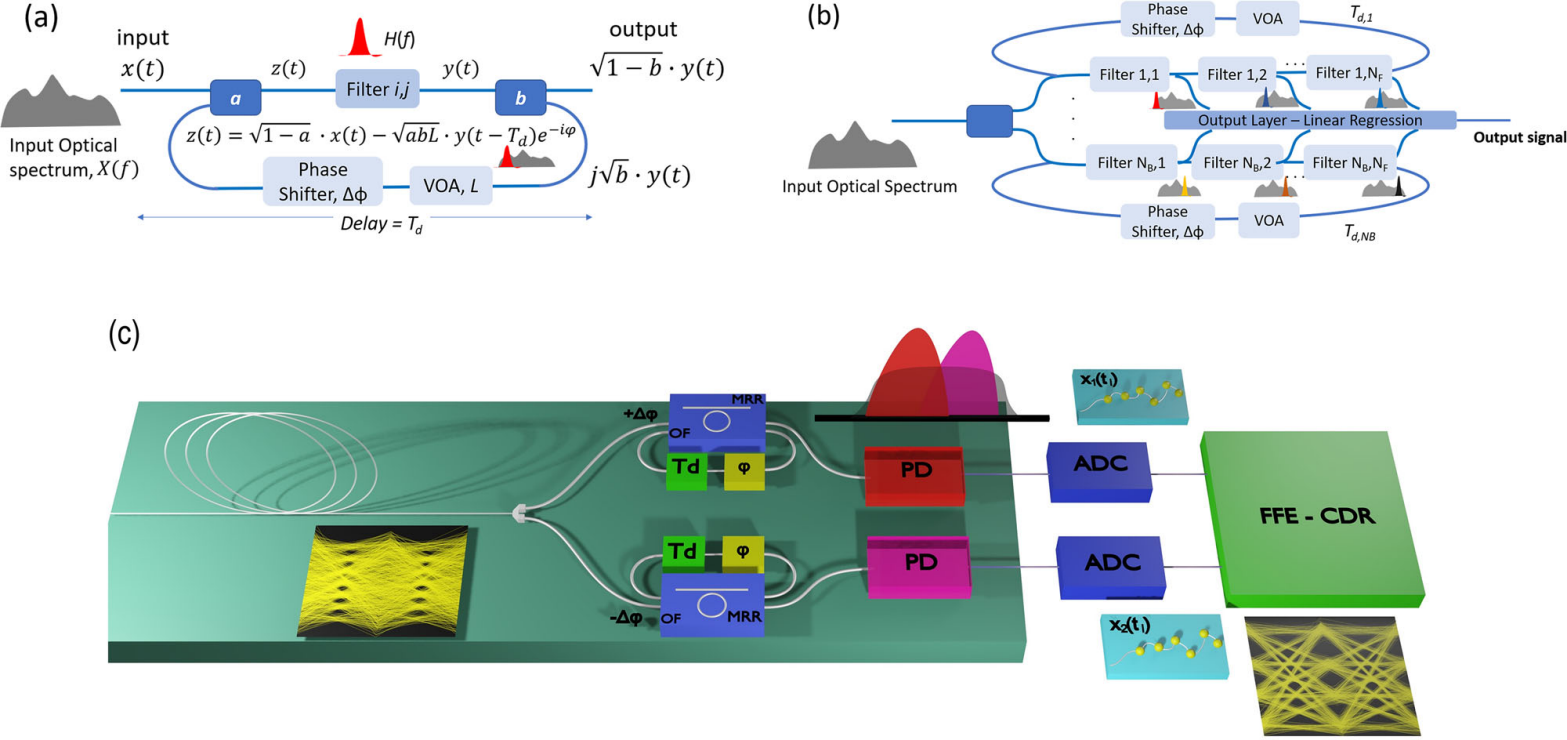
The architectural structure of Recurrent Optical Spectrum Slicer-Neural Network (ROSS-NN). **a** Configuration of a single ROSS-NN node, (**b**) The architecture of ROSS-NN consisting of *N*_*B*_ filter banks with each bank consisting of *N*_*F*_ filters in-a-loop serving as recurrent optical spectrum slicers. **c** The architecture of ROSS-NN as a hardware neuromorphic processor for high-speed optical communications signals suffering from chromatic dispersion, bandwidth limitations of the transceiver and nonlinear effects.

The basic unit of our system is a recurrent node consisting of a first-order bandpass or bandstop optical filter, two couplers and a feedback loop with delay *Τ*_*d*_. The feedback loop is equipped with a phase shifter so as to adjust the feedback phase, whereas feedback losses (*L*) can be adjusted during fabrication or through the optional inclusion of a variable optical attenuator (Fig. 1a)^31^. The whole architecture can be monolithically integrated using mature silicon photonics technology, whereas the optical filters in the loop may be implemented by means of Mach-Zehnder Delay Interferometers, MRRs or any equivalent bandpass/bandstop optical filter. In Fig. 1b, we present a generic architecture consisting of multiple recurrent optical filters organized in separate filter banks, spectrally slicing different frequency bands of the input optical signal. This complex architecture can be easily implemented if add/drop MRRs are used, as rings can be interconnected using through ports and provide outputs using drop ports which are directed to the output layer. The output layer could be implemented in the optical domain, by combining filter’s outputs through an optical combiner, followed by a single photodiode and analog-to-digital converter (ADC)^32^, or in the digital domain with the use of a photodiode/ADC per filter output. Depending on the problem to be solved, the architecture may contain one or more filters incorporated in one or multiple loops. The number of filters or loops is mostly limited by optical losses and the corresponding signal-to-noise ratio at the output layer. Each recurrent node focuses on a specific frequency band of the input optical signal. Thus, the number of nodes is on one hand related to the required granularity of spectrum slicing as dictated by the problem and should on the other hand be sufficient to properly cover the full optical bandwidth to be processed.

The transfer function of the recurrent node in Fig. 1a is given by:

1 $${H}_{{node}}(f)\,=\,\frac{\sqrt{1-a}\,\sqrt{1-b}H\left(f\right)\,}{1+\sqrt{a\,{bL}}\,H\left(f\right)\,{e}^{-i\left(2\pi f{T}_{d}+\varphi \right)}}$$

where *a* and *b* are the coupling ratios at the input and output, *L* the variable optical attenuator induced losses, *T*_*d*_ is the total delay of the loop. *H*(*f*) is the transfer function of the in-loop filter(s) and *φ* is the phase imposed by the in-cavity phase shifter. The aforementioned nodes can be considered as building blocks of ROSS-NN which may serve as a photonic RNN or an RC. In particular, we have the possibility to follow the RC paradigm and mimic random inter-node connectivity by stochastically varying the complex amplitude of the signal injected from filter to filter in Fig. 1b. To further enforce random connectivity, we can induce arbitrary frequency offsets between adjacent nodes that contribute to a stochastic mixing of the frequency components handled by successive filter nodes belonging to the same bank. In a RC-like treatment of the configuration depicted in Fig. 1b, we follow the RC-related training, thus restricting training only at the output layer of Fig. 1b. On the other hand, one may handle all these variables (filter bandwidth, offset between successive filters, phase shifter, feedback attenuation etc.) as hyper-parameters that can be optimized for a specific task. In this case, the network mostly resembles an RNN configuration where optical weighting between units can be applied in different forms (variation of signal amplitude and phase after each filter, variation of frequency offset between adjacent filters). Figure 1c depicts the scheme that has been numerically tested and that provides improved results in the most important application of transmission impairments mitigation at very high baud rates (>100 Gbaud) and at low complexity. It will be shown in the results section that this simple scheme relying on two passive recurrent optical filters for PAM-4 and three filter for 16-QAM has the ability to outperform state-οf-the-art digital equalizers, while its complexity and therefore its energy footprint is over an order of magnitude lower.

### ROSS-NN for the nonlinear autoregressive moving-average (NARMA) task

One of the key properties that a recurrent neuromorphic scheme should be able to address is the reproduction or prediction of pseudo-chaotic sequences with increased temporal complexity. Although these tasks (NARMA, Santa Fe, Mackey-Glass etc.) are of minor importance application-wise, their successful processing can assess the overall efficiency of a neuromorphic dynamical scheme. We chose the NARMA task, originally introduced in^33^. In this context, we utilized a pseudo-random input, drawn from a uniform distribution *u*(*n*) and computed the tenth order NARMA-10 sequence *y*(*n*).

2 $$y\left(n+1\right)=	\; 0.3\cdot y\left(n\right)+0.05\cdot y(n)\left[{\sum }_{i=0}^{9}y(n-i)\right] \\ 	 +1.5\cdot u\left(n-9\right)\cdot u\left(n\right)+0.1$$

**Fig. 2:**
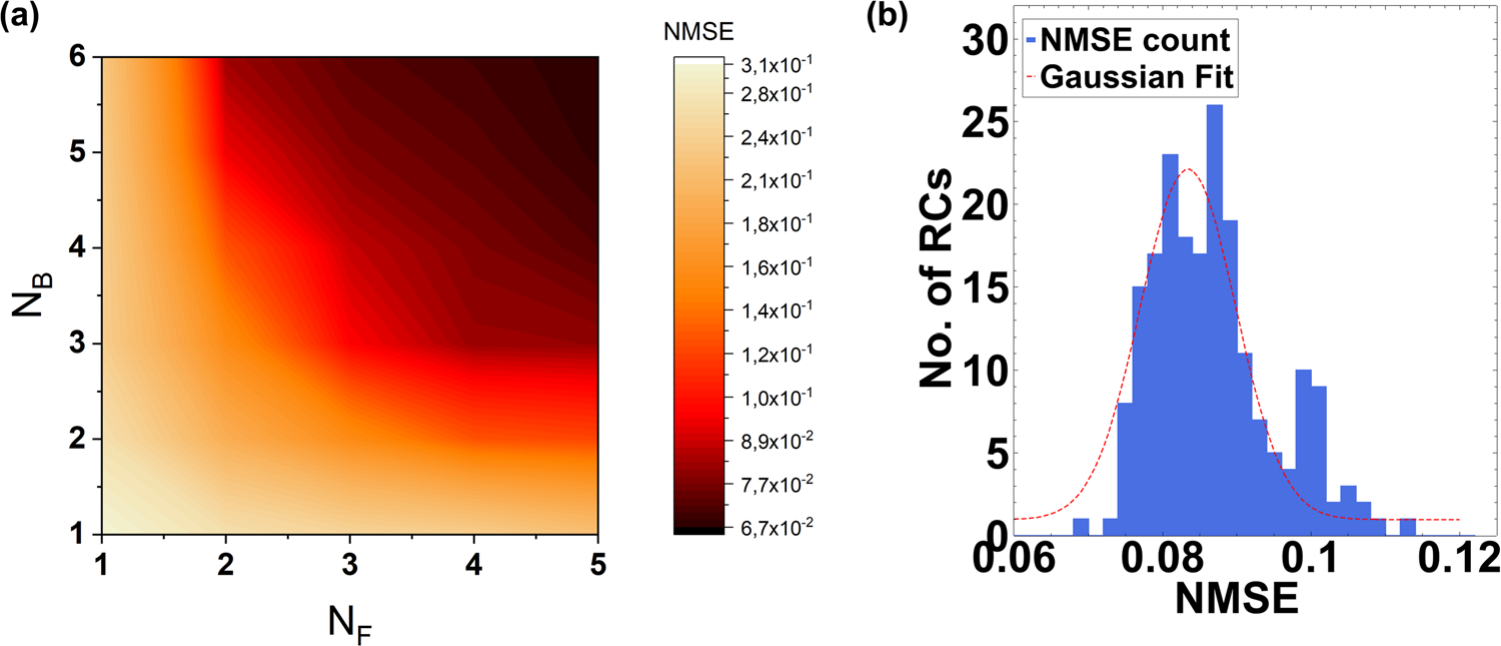
Results of for the Nonlinear Autoregressive Moving-average (NARMA)-10 reproduction. **a** Normalized Mean Square Error (NMSE) as a function of the number of filters per bank (*N*_*F*_) and the number of filter banks (*N*_*B*_) in the Recurrent Optical Spectrum Slicer-Neural Network (ROSS-NN) configuration. The actual number of spatial nodes is equal to *N*_*F*_ *x N*_*B*_. **b** Distribution of NMSE values for random implementations of ROSS-NN internal connectivity in the form of random frequency offset of all filters’ central frequency.

Each value from the pseudo-random input sequence *u*(*n*) is used to modulate the amplitude of a laser source at a rate of 40 Gbaud. The optical input is equally split and injected to a ROSS-NN configuration consisting of one to six banks (*N*_*B*_), where each bank embeds one to five filters (*N*_*F*_) that are numerically implemented as add/drop MRRs. Intra-cavity losses and the coupling coefficient between the circular and straight waveguides of the MRRs have been used as hyperparameters to tune the Q-factor and bandwidth of each MRR. The central frequency of each filter bank can be easily adjusted in a course way by placing a phase tuner inside the MRR. The drop ports from all filters are considered as the scheme’s optical outputs and are fed to a typical detection scheme (photodiode and ADC) followed by a digital linear regression as the RC’s trainable output layer. The regression has 10 taps, matching the NARMA’s order. The purpose of the system is to train the output layer of the RC so that the system correctly emulates the sequence *y*(*n+*1) after training. To achieve this, 50% of the NARMA outputs of 4000 symbols was used as a training sequence, regulating the weights of the linear regression. Following the training procedure, the RC was fed with 2000 *u*(*n*) subsequent symbols and the generated output $\hat{y}(n+1)$ was recorded. Accuracy was evaluated by computing the normalized mean square error (NMSE) between *y*(*n+*1) and $\hat{y}(n+1)$. Taking into account that ROSS-NN is based on the spectral slicing property, for each combination of banks/filters, all the critical parameters such as the MRR’s *Q*-factor, the detuning of each bank relevant to the signal’s bandwidth and the central frequency of each MRR resonance compared to the banks center, were scanned so as to achieve optimum performance. In Fig. 2a, it can be seen that in order to get NMSE < 0.1, a minimum of number banks equal to *N*_*B*_ = 3 each having *N*_*F*_ = 4 MRRs is needed, resulting to only 12 physical nodes. A critical observation is that for each neural configuration (*N*_*F*_*, N*_*B*_) each filter’s bandwidth and each bank’s spectral band is optimized so that the full spectrum of the incoming signal is covered. Up to now we did not really treat the ROSS-NN as a RC network. Although we restricted training in the linear regression part, we also tried to optimize all the other hyperparameters related to the number of filters per bank, the number of banks and the exact shape in terms of bandwidth and central frequency of each individual MRR. This treatment contradicts with one of RC’s most fundamental aspects, the randomness of connections, that contributes to its hardware friendliness. In order to evaluate the impact of randomness on performance, we have assumed realistic structural deviations, as if this scheme was realized in a typical silicon photonic platform^34,35^ (methods). In particular, we solved the same NARMA task using 200 ROSS-NN instances (RCs in Fig. 2b) each having *N*_*B*_ = 5, *N*_*F*_ = 5. These ROSS-NN instances exhibit structural deviations compared to an ideal prototype, such as the effective refractive index of each MRR due to waveguide roughness, resulting to frequency detuning deviations, inter-MRR transmission coefficient and delay (phase). The ideal in our case is a ROSS-NN whose key properties (detuning, bandwidth) are optimized for the specific task as hyperparameters and no fabrications-related imperfections are considered. In Fig. 2b, the histogram for this scenario alongside a gaussian fit is presented showing that the $\overline{{NMSE}}=0.086\pm 0.0005$, not deviating significantly from the ideal NMSE. The NARMA results obtained from the ROSS-NN scheme can be directly compared to other numerical RC investigations that offer NMSE values in the same order using different RC numerical implementations of over 50 virtual nodes^14,36^. It is reminded that a linear shift register can provide NMSE~0.16^13^. Hence, while preserving marginal power consumption and integration capabilities, ROSS-NN presents good performance along with the reduction in node count compared to the state of the art. Furthermore, the proposed scheme can address this task without any speed penalty that is present in time-delayed RCs, whereas even higher bandwidths can be envisioned without any additional considerations apart from signal-to-noise ratio, the elevated analog bandwidth of the photodiodes and the ADC, which are typical limitations for all photonic neuromorphic or signal processing schemes in general. Lastly, it is worth mentioning that the previous demonstration showcased that ROSS-NN is capable of playing the role of a general-purpose recurrent processor in the well-established NARMA-10 test. However, by solely modulating the amplitude of a carrier, one cannot fully harness the true capabilities of a system offering coherent processing. In the next section, a real-world problem requiring coherent processing and frequency diversity is considered in order to reveal all the merits of ROSS-NN proposition.

### Few-node ROSS-NN as a photonic hardware accelerator in 100 Gbaud and beyond optical communication systems

Until today, data center interconnects are mainly based on cost-effective direct detection systems covering distances from 500 m to 80 km. The main limitation in such distances is the interaction between CD and the square-law of the photodiode which results in power fading. Generically, a real-valued unipolar signal at the transmitter with a direct current bias can be expressed as:

3 $$r\left(t\right)=\,s\left(t\right)+\,c\left(t\right)$$

in which *s*(*t*) is the original signal and *c*(*t*) is the optical carrier related to direct current bias. The received signal with square-law detection is represented as:

4 $$y\left(t\right)=	\; {c}^{2}\,\left(t\right)+\,{\left|s\left(t\right)\otimes h\left(t\right)\right|}^{2}\,+\,2\,c\left(t\right)\,\cdot s\left(t\right)\\ 	 \otimes \,{F}^{-1}\big[{Re}\big\{{H}_{{fiber}}(\;f\,)\big\}\big]$$

where ⊗ is the convolution operator, and

5 $${Re}\big\{{H}_{{fiber}}(\,f\,)\big\}={\cos }\big({2\pi }^{2}{{\beta }_{2}}^{2}L{f}^{2}\big)$$

in which *β*_2_^2^ is the second order dispersion coefficient, *L* denotes fiber length and *f* represents signal frequency. Based on Eq. (5), it can be found that the received signal suffers from power fading caused by CD and its nonlinear transformation at the photodiode. This dispersion-induced power fading will result in deep spectral zeros when ${2\pi }^{2}{{\beta }_{2}}^{2}L{f}^{2}-\frac{\pi }{2}$ is a multiple of *π*.

Many works in the literature have been devoted to the mitigation of this distortion and a number of techniques such as optical dispersion compensation, Single Sideband modulation, digital equalization in the form of Decision Feedback Equalizer^37,38^ or Maximum Likelihood Sequence Detectors (MLSD)^39,40^ have been reported. The quadratic dependence of CD on baudrate is the reason why next-generation 112 Gbaud IM/DD links are forced to rely on heavy digital signal processing (DSP) algorithms that cancel out accumulated dispersion up to 10 km, while for longer links, coherent detection is the only viable, but expensive solution. ROSS-NN is capable of equalizing both IM/DD and coherent schemes.

**Fig. 3:**
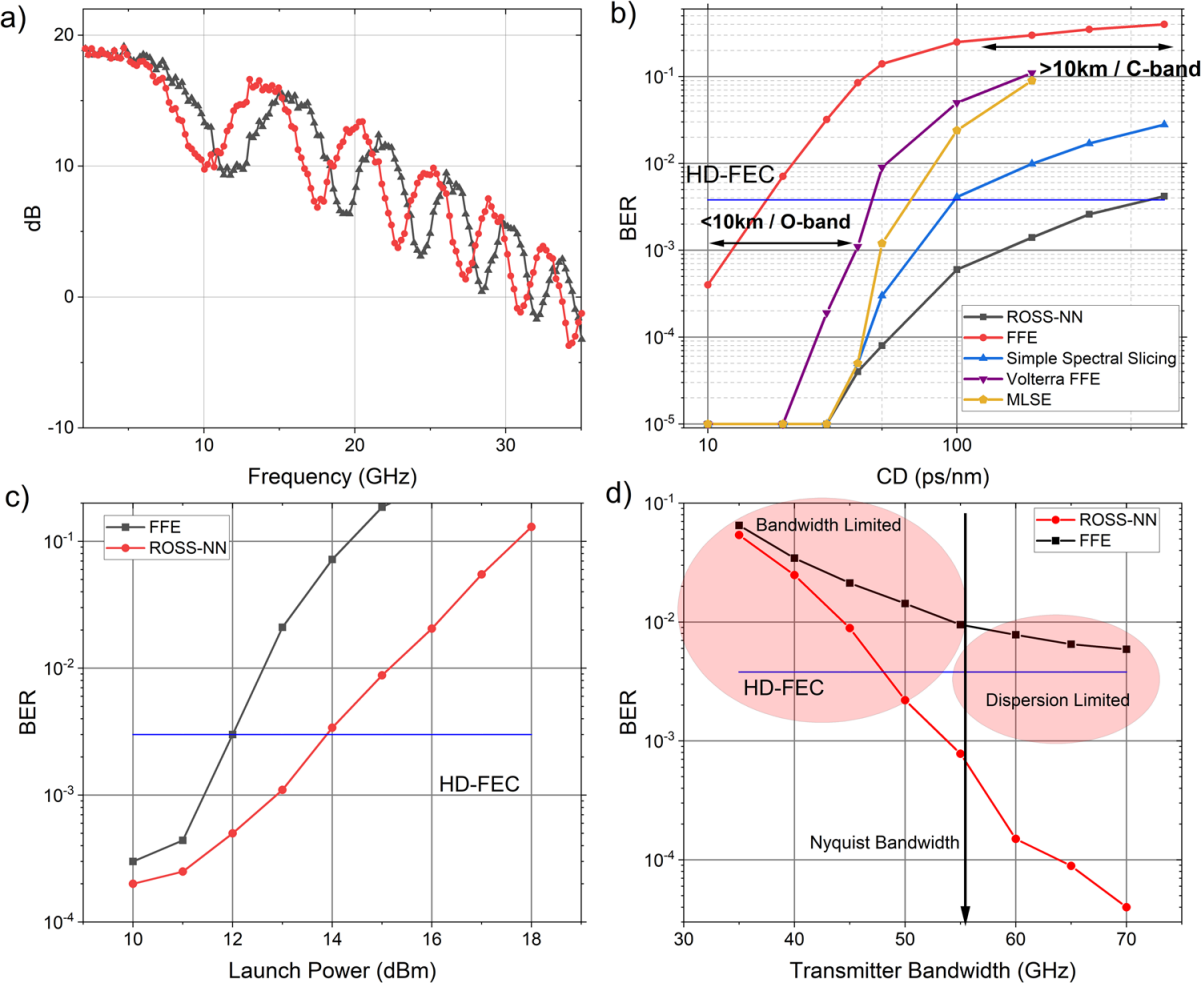
Performance of Recurrent Optical Spectrum Slicer-Neural Network (ROSS-NN) in the mitigation of transmission impairments in intensity-modulated optical communication links. **a** The spectral response of the recurrent nodes after photodetection. The power fading effect, due to 20 km C-band transmission, is observed with multiple spectral dips in both outputs, however, frequency diversity is also observed. With the proper adjustment of bandwidth, frequency detuning and delay values characterizing the ROSS-NN system, we can almost completely eliminate power fading and provide additional memory for tackling intersymbol interference caused by Chromatic Dispersion (CD). **b** The CD tolerance of the proposed RC-system in comparison with a system exploiting two filters without any feedback and Feed Forward Equalizer (FFE), Maximum Likelihood Sequence Estimator (MLSE), Volterra Non Linear Equalizer (VNLE) as postprocessing. While for small accumulated CD values (for example, <20 km reaches in the O-band), two simple filters or heavy MLSE, VNLE can achieve acceptable results, for higher CD values, only the system with the recurrent nodes can achieve results below Hard Decision - Forward Error Correction (HD-FEC) limit. **c** The efficiency of the proposed system in the mitigation of Kerr-related non-linearities when CD is optically compensated. The system provides 2 dB gain in comparison with a linear algorithm. **d** The performance of the ROSS-NN-system and the FFE as a function of transmitter’s bandwidth for fixed 35 GHz bandwidth per photodiode. The results refer to a 40 km O-band link with a group velocity dispersion parameter D = 0.5 ps nm^−1^ km^−1^.

We first demonstrate that ROSS-NN consisting of two nodes is capable of mitigating transmission impairments in an IM/DD link, at the challenging next generation 112 Gbaud rates, achieving even 60 km reach with very high CD tolerance. In general, an intuitive way to understand RC operation, is as a nonlinear dynamical system that acts as a pre-filter on the input data, transforming it into a higher dimensional space^41^. This is achieved by using a transformation resulting in multiple outputs which have undergone different routes in the spatial, temporal and – most importantly in our case – the spectral domain. Gonon et al.^42^ clearly state that very important features of causal and time-invariant filters like the fading memory property or universality are naturally inherited by reservoir systems. Our proposition consists of nodes that are causal and time-invariant optical filters that provide fading memory as shown in Supplementary Discussion 3 and that have the echo state property, since the reservoir will asymptotically wash out any information from initial conditions due to the passive nature of the recurrent filter (optical feedback below 1) which precludes instabilities in the dynamical behavior. The recurrent connectivity offers rich and frequency-dependent memory (see Supplementary Discussion 3) which is important when transmission impairments are caused by nonlinear channels with memory, such as single-mode fibers. The proposed ROSS-NN consisting of two recurrent nodes (Fig. 1c) provides evident frequency diversity of power fading, characterizing the distorted signals at the two outputs due to CD (see Fig. 3a). This is achieved by treating differently the lower and higher frequency components through spectral slicing of each sideband and by leveraging optical feedback as an extra mechanism to enhance specific frequency components and fading memory. Both outputs are followed by photodiodes with bandwidth lower than the baud rate and ADCs that require only one sample per symbol (sps), thus showing that real-life implementation of the scheme is practical at high baud rates (>100 Gbaud) and more appealing than coherent detection which requires at least 1.25 samples/symbol in order to decode the signal^43^. A feed-forward equalizer (FFE) follows the ADC in order to act as a linear regression stage and to assist in the elimination of Inter-symbol Interference and bandwidth limitations caused by CD and transceiver optoelectronic components. As for the accumulated CD tolerance, Fig. 3b compares the proposed system with a system consisting of two simple optical filters offering frequency diversity^22^ and an FFE, showing that, especially for high CD values as the ones in C-band, and for reaches beyond 10 km, ROSS-NN is the only viable solution with one order of magnitude better BER performance. Furthermore, the ROSS-NN outperforms state-of-the-art digital algorithms like MLSE with 5 taps and a 3^rd^ order VNLE with 91,31,11 taps for each order (Fig. 3b). ROSS-NN, compared to VNLE or MLSE, can perform superior equalization, requiring only 40-100 multiplications/bit at the digital back-end, whilst the Volterra algorithm employed here requires over 2400 and the heavy MLSE of 5 taps over 10000, for the results presented in Fig. 3b. It must be stressed that, in the strict environment of short-reach communications, the power consumption is of paramount importance. With a two-node ROSS-NN, we propose a receiver that, apart from its almost passive optical part (sub-μW consumption for state-of-the-art phase shifters^44^), consumes less than 1 W for 112 Gbaud, based on the latest 7 nm FinFET technology^45,46^ for the two relaxed 40 GHz ADCs and a 50-tap FFE. For comparison, a light 2-tap MLSE with 128 multiplications, with a 56 GHz ADC, would consume over 1.5 W. In a transceiver with four coarse wavelength division multiplexed channels, this corresponds to more than 2 W or 20% reduction in the transceiver power envelope.

In order to benchmark the RC-system in a harsh nonlinear transmission environment, we compensate CD using a dispersion compensating fiber with a high non-linear parameter and launch powers in the numerical model that excite Kerr non-linearities. By comparing ROSS-NN with a simple FFE as a postprocessing method, 2 dB higher tolerance in nonlinear effects is achieved (Fig. 3c). ROSS-NN is also capable of mitigating bandwidth limitations induced by optoelectronic components of the transceiver. The bandwidth provided by vendors of Mach-Zehnder modulator drivers and Digital-to-analog converter is in the order of 60 GHz, which poses strict limitations to the extension of baud rate beyond 112 Gbaud. Although FFEs and Pre-Emphasis filters are recognized tools for the mitigation of this distortion, it still constitutes a major problem. In Fig. 3d we provide results for the tolerance of the proposed system to the limited bandwidth of the transmitter. Τhe photodiode bandwidth after each node is assumed constant at 35 GHz. It is shown that even with 50 GHz analog bandwidth in the transmitter, sub-Hard Decision-Forward Error Correction results could be achieved even for a 40 km long O-band link. If the 25% overhead Soft Decision Forward Error Correction is considered, the analog bandwidth could be reduced to almost 45 GHz. It must be stated that all critical hyperparameters of the ROSS-NN have been optimized in this study (see Supplementary Discussion 2).

**Fig. 4:**
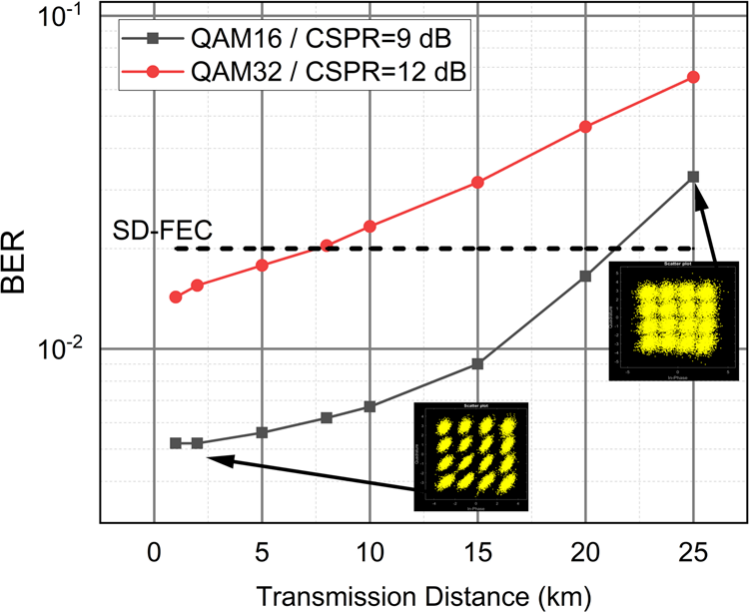
Performance of Recurrent Optical Spectrum Slicer-Neural Network (ROSS-NN) in the extraction and equalization of coherent signals. Bit-Error Rate results as a function of transmission reach for Quadrature Amplitude Modulation (QAM)-16 and QAM-32 in the self-coherent configuration. With the QAM-16 format, 20 km of transmission is achieved with carrier to signal power ratio (CSPR) of 9 db, while with QAM-32 the reach is at least 5 km with a CSPR of 12 dB. The transmission reach is compared with the Soft Decision-Forward Error Correction (SD-FEC) limit 2 × 10^−2^.

We further benchmarked a three-node system in a coherent transmission link in order to prove the versatility of the ROSS-NN and its suitability to deal with coherent modulation formats. We take advantage of a residual carrier that permits the reception of the coherent signal with simple photodiodes following the paradigm of cost-efficient self-coherent systems^47^. State-of-the-art 120 Gbd QAM-16 and QAM-32 scenarios are numerically simulated solely focusing on chromatic dispersion mitigation with the use of ROSS-NN. In this baudrate, 400 Gbps and 500 Gbps net data rates can be achieved in a single wavelength and polarization. We keep the Carrier-to-Signal Power Ratio within the limits of a typical Kramers-Kroning receiver^47^, namely between 9 and 12 dB. In such systems, the CD effect is linear if coherent detection is utilized. In this work we choose the much simpler direct detection leveraging the residual carrier, however, dispersion management without using the efficient but computationally heavy Kramers Kronig algorithm, constitutes a rather challenging signal processing problem. By employing ROSS-NN, we perform phase-to-amplitude conversion that maps all the different QAM symbols to the amplitude domain. Spectral slicing by three nodes also relaxes the need for large analog bandwidth in the order of 40-45 GHz, thus constituting an attractive solution in the bandwidth-hungry area of coherent communication technology. The readout is split in two linear layers, one per quadrature. In Fig. 4, indicative results of the BER performance of the two modulation formats is presented as a function of transmission distance in an O-band link. Sub Soft Decision- Forward Error Correction performance is achieved after 20 km using 16-QAM with a Carrier-to-Signal Power Ratio of 9 dB while almost 8 km reach is achieved with 32-QAM and Carrier-to-Signal Power Ratio of 12 dB. Thus, we present a simple direct detection scheme suited for M-QAM at high baud rates based on a very simple DSP at the back-end of the receiver. It must be also stated out that the BER tolerance to transmitter phase noise is very high (see Supplementary Fig. 6a). The results depicted in Fig. 4 consider transmitter linewidth in the order of 300 kHz, whilst coherent receivers require narrow linewidth lasers (< 50 kHz).

Finally, in Table 1, the proposed architecture is compared with numerical results from similar works in the literature in terms of the well-established metric of bitrate-distance product. The comparison reveals that in the inherently coherent and frequency depended task of dispersion compensation in high baud rate optical transmission, recurrent coherent processing provides the best performance over all state-of-the-art solutions at only 1 sps.

**Table 1:** Comparison of different Reservoir Computing works for equalization of optical links in terms of bitrate-distance product.

Work	Rate/Format	Distance (L)	bitrate*L (Gbps*km)	Analog/Digital (samples per symbol)
^20^	28 Gbd/PAM4	27 km	1512	8
^62^	40 GBd/OOK	15 km	600	–
^29^	32 GBd/OOK	80 km	2560	2
[this work]	112 GBd/PAM4	80 km	17920	1

Format refers to pulse amplitude modulation (PAM) and on-off keying (OOK).

## Discussion

In this paper we have proposed and numerically evaluated a neuromorphic photonic concept based on recurrent optical spectrum slicing, implemented by optical filters embedded in a delay loop. Such a concept constitutes a practical and realization-ready solution in silicon photonics chips^48^ or even leveraging programmable photonics platforms^49^. The main advantage of the proposed scheme is its compatibility with direct processing in the optical frequency domain, thus rendering the specific neuromorphic approach suitable for spectral decomposition and processing of ultra-broadband signals (~ THz). Especially, when ultra-fast processing is necessary, a solution that can easily scan and process broad optical spectra directly in the optical domain and with minimum power consumption or need for data storage is a useful tool. ROSS-NN can cover this need and play the role of a high-speed optical frequency processor in applications such as high throughput real-time flow-cytometry^50^, high-resolution 3D imaging^51^ and generally in tasks where simultaneous spectro-temporal knowledge is required at very fast rates.

It must be stressed that, by introducing the spectral degree of freedom in addition to the existing time and space ones, ROSS-NN outperforms both time-delayed and spatially distributed RC systems in processing speed capabilities. In the case of time-delayed systems, for processing a signal of bandwidth *B*, a system of *N* virtual nodes requires sampling bandwidth *R* equal to *N* x *B* both at the input and at the output. A spatially distributed system requires *N* receivers of *B* bandwidth, so the total *R* equals again to *N* x *B*, while a ROSS-NN requires *N* receivers of bandwidth slightly greater than *B*/*N* due to slicing, or a total *R* ≈ *B* in the general case. Furthermore, even in the case of a modulated carrier, where the filtering cannot be conducted far away from carrier frequency, spectral slicing still relaxes the required receiver bandwidth.

Since optical communications industry has the strongest foothold in photonic applications for real-life problems, we anticipate that ROSS-NN could have an impact in the advent of edge-cloud interconnects. Edge-cloud era seeks for straightforward, low-cost ideas for facing the strict requirements in low latency, high bandwidth, stability and power efficiency. Already, moving digital processing as near as possible in the optical transceiver, through co-packaged optics is a colossal migration step, which will disrupt the field in the next decade^52^. But, implementing computing and processing directly in the optical domain, in the core of the optical engine, is an ambitious endeavour. By relaxing the optical bandwidth requirements (less than 40 GHz optoelectronic components for 112 Gbaud and beyond signals as shown in Figs. 3 and 4) and keeping DSP to the bare minimum, through optical pre-processing, ROSS-NN could counteract the severe power consumption issues that 800 G technology poses, creating even 20% savings in the 10–40 km transceivers. In comparison with coherent technology, which conquers even the shortest reach scenarios of Inter Datacenter Communications^53^, ROSS-NN offer multi-Watt reduction in the overall transceiver power budget, as 800 G coherent modules are anticipated to have over 20 W power dissipation^54^, while with the proposed hardware accelerator we estimate less than 14 W. Combating CD even in Extended Reach (ER) 40 km channels with relaxed energy consumption, ROSS-NN receivers constitute an appealing tool for the 6 G, Internet-of-Everything and Industry 4.0 revolutions of the next years, either in the IM/DD or in its self-coherent approach.

An interesting field towards further exploration of ROSS-NN systems is their training. As already pointed out, ROSS-NN can be used as building block for both RC and RNN implementations. In the former case, the readout layer is one of the most critical parts of the architecture. Digital or even optical readout should be studied in-depth in forth-coming studies. First evaluations show that optical readout further enhances the performance of the network in specific tasks from the telecom arena, due to the fact that the nonlinear activation is boosted when all favored frequency components from diverse nodes are combined on the same square law detector^32^. When the network is operated as an RNN, then activities on training become even more demanding as all hyperparameters along with the readout layer must be optimized concurrently utilizing back-propagation or equivalent techniques. Another critical property of ROSS-NN attributed to its recurrent nature is that ROSS-NN has the property to enhance the number of output traces through time multiplexing which is equivalent to temporal unfolding of each spatial node’s dynamical behavior^55^, or to a post-FIR filter that expands the readout layer^56^. More complex networks can be realized if the characteristic delay of the loop of each bank is varied. ROSS-NNs open new paths in investigating and training neural networks in the frequency domain and could be also considered as a neuromorphic approach even in the electronic domain where the implementation of analog filters with diverse transfer functions is mature and CMOS technology permits the hardware implementation of complex networks consisting of thousands of filter nodes with fine granularity.

## Methods

### Recurrent node simulation

In this work, we propose a recurrent filter node for RNN/RC architectures. Such recurrent filters are easily integrated into photonic circuits along with many other optical components like semiconductor optical amplifiers, variable optical attenuators, phase shifters, couplers etc.^57,58^. In our simulations, each filter is modelled through its transfer function *H*(*f*), while the phase shifters and variable optical attenuators are inserted as phase and feedback terms in (1).

The transfer functions of the Mach-Zehnder Delay Interferometers and MRR filters *H*(*f*) are given by

6 $${H}_{{MZDI}}\left(f\right)=\frac{1}{2}\,\left[1+{e}^{-i2\pi \left(f-{f}_{0}\right)\Delta T}\right]$$

where *f*_0_ is the central frequency of the filter and Δ*T* is the delay difference between the two arms. *f*_0_ can be tuned with the use of phase shifter in one of the two Mach-Zehnder Delay Interferometers arms. Regarding MRRs, the through port and the drop port transfer functions are given by (7)–(8),

7 $${H}_{{MRR},{Through}}\left(f\right)=\frac{-\,{{T}_{2}e}^{\varPhi }\,+{T}_{1}\,-\,({{K}_{1}}^{2}\,{T}_{2}\,{e}^{\varPhi })\,}{(1-{T}_{1}\,{T}_{2}\,{e}^{\varPhi })}$$

8 $${H}_{{MRR},{Drop}}\left(f\right)=\frac{-{K}_{1}{K}_{2}\,{e}^{\varPhi }}{(1-{T}_{1}\,{T}_{2}\,{e}^{\varPhi })}$$

9 $$\varPhi =\frac{-a\,L/2\,-\big[i2\pi \,\big(\,f-{f}_{0}\big)\,L\,{n}_{{eff}}\big]}{c}$$

where *T*_*1*_*, Τ*_*2*_ the transmittance, *K*_*1*_*, Κ*_*2*_ the coupling coefficientσ, *a* the waveguide losses, *n*_*eff*_ the effective refractive index, *L* the circumference of the ring and *c* the speed of light.

### Transmission system simulation – ROSS-NN evaluation in optical communication tasks

The transmission system consists of a semiconductor laser modelled with the well-known Lang-Kobayashi rate equations^59^ for the complex slowly varying amplitude of the electrical field *E*(*t*) and the carrier number inside the cavity *N*(*t*).

10 $$\kern0.9pc \frac{{dE}}{{dt}}=\frac{1+i\alpha }{2}\left[{G}_{s}-\frac{1}{{t}_{{ph}}}\right]{E}_{{{{{{\rm{f}}}}}}}+\sqrt{2\beta N}\xi$$

11 $$\frac{{dN}}{{dt}}=\frac{I}{q}-\frac{N}{{t}_{n}}-G{\left|E\right|}^{2}$$

12 $$G=\frac{g\left[N-{N}_{0}\right]}{1+s{\left|{\rm E}\right|}^{2}}$$

Here *α* is the linewidth enhancement factor, *g* is the gain parameter, *s* is the gain saturation coefficient, *t*_*ph*_ is the photon lifetime, *t*_*n*_ is the carrier lifetime and *N*_*0*_ is the carrier number at transparency. The simulation values of these parameters are given in Table 2.

**Table 2:** Numerical Model Parameters.

Symbol	Parameter	Value
g	Differential gain parameter	1.2 × 10^−8^ ps^−1^
s	Gain saturation coefficient	5 × 10^−7^
β	Spontaneous emission rate	1.5 × 10^−10^ ps^−1^
tn	Carrier lifetime	2 ns
N_0_	Transparency Carrier Number	1.5 × 10^8^
A	Linewidth enhancement factor	3
ω_0_	Central oscillation frequency	1.206 × 10^15^ rad s^−1^
I	Bias current	35 mA

The symbols for the laser modulation rely on the Mersenne Twister pseudo-random generator with a unique seed and a repetition period of 2^19937^-1. The reason for this measure is to hinder ROSS-NN from anticipating the next symbol in the sequence and thus, overestimate the equalization results. An external Mach-Zehnder modulator is assumed, acting as a 2^nd^ order Butterworth filter, emulating bandwidth limitation at the transmitter. We simulate, with the integration of Nonlinear Schrödinger equation using the Split-step Fourier method, the transmission of 112 Gbaud PAM-4, QAM-16 signals in a range of 10 km to 60 km transmission distances. Signal propagation in our model is governed by Manakov equations^60^. The group velocity dispersion parameter takes values between *D* = 0.5 ps nm^−1^ km^−1^ and *D =* 4 ps nm^−1^ km^−1^ for O-band transmission, while *D*= 17 ps nm^−1^ km^−1^ is assumed for simulations in the C-band. The fibre losses are set to *a* = 0.34 dB km^−1^ in O-band and *a* = 0.21 dB km^−1^ in C-band. The non-linear parameter is *γ* = 1.3 W^−1^ km^−1^, while when dispersion compensation fiber is used, its *γ* = 6 W^−1^ km^−1^. In the receiver side, a pre-amplifier with 5 dB noise figure is simulated in order to compensate for the transmission losses, the chip’s insertion loss and the initial splitter. In a real-life scenario, a semiconductor amplifier in single wavelength transmissions or a Doped Fiber Amplifier in wavelength division multiplexing scenarios could play the role of pre-amplifier. The intensity of recurrent nodes output is captured with photodiodes modelled as a square-law element of responsivity *R* = 0.8 A/W and bandwidth 35 GHz. Shot and thermal noise are taken into account. An 8-bit, 112 Gs/s ADC follows each photodiodes, with analog bandwidth of 35 GHz.

### Training of the readout layer

The ADC generates one sample per incoming symbol. These digital samples are inserted to a linear classifier that resembles the typical symbol-spaced FFE block in IM/DD DSP. The length of the FFE is adjusted so as to match the channel’s memory which is proportional to the group delay time *Τ* = *D Δλ L*_*D*_, where *D* is the second order dispersion parameter, *Δλ* the optical bandwidth occupied by the signal and *L*_*D*_ the transmission distance. In these simulations the memory ranges from 11 to 21 symbols for O-band transmission, while for C-band links this number reaches up to 71 symbols. Half of the symbols are considered as pre-cursor and half as post-cursor taps. The weights, *b*, of the linear equalizer are calculated by finding the pseudo-inverse matrix through Tikhonov regularization. With 20000 symbols for training and 100000 symbols for testing, we achieve enough precision for BER above 10^−4^. When QAM is considered (Fig. 4), we apply two separate linear readouts, one for the real part and one for the imaginary part (see Supplementary Discussion 2).

### NARMA10 task

The pseudo-random signal that is used as input for the NARMA sequence consists of 4000 samples drawn from a uniform distribution (python’s generator). The values range from 0 to 0.5. The NARMA output is computed assuming to have an order (memory) of 10. Simulation wise, the pseudo-random values are oversampled using 8 samples per symbol and the time scale was regulated so as to result to a rate of 40Gsymbol/sec. These analog values were used to amplitude modulate a continue-wave (CW) laser with power of 0 dBm, assuming an extinction ratio of 20 dB. The optical signal was assumed to be amplified, using an amplifier gain of 10 dB and a noise figure of 5 dB. The signal was subsequently split according to the number of ROSS-NN nodes used. In this case inside each ROSS-NN module, add/drop MRR filters are assumed. The outputs from the MRR’s drop ports were recorded by photodiodes and typical shot and thermal noise was incorporated. The computed photocurrents were normalized and they were fed to a linear regression algorithm with 10 taps so as to match the NARMA’s memory. 2000 samples of the NARMA were used as teacher so as to train the weights of the linear regression. Following this step, 2000 samples from NARMA’s pseudorandom input were fed to the ROSS-NN for inference; aiming to reproduce the actual NARMA output. The two traces (predicted and reproduced) were compared using the normalized mean square error.

Regarding the neuromorphic architecture used for addressing NARMA, we varied the number of banks (ROSS-NN node) and MRRs per bank. For all instances the MRRs were assumed to have a radius of 55 μm and propagation losses of 0.4 dB cm^−1^. The waveguides connecting the MRRs per bank were assumed to exhibit transmission coefficient of 0.95, whereas inter-MRR delay was fixed at 0.1 of symbol duration. The detuning of each MRR was assumed to be such that the combination of banks and filters span over the whole bandwidth of the signal. Therefore, the center frequency of each MRR and the spacing among different filters was scanned for each combination of banks and filters per bank. In the same context, the coupling coefficient for each MRR, partially regulated filters bandwidth and was scanned so as to locate the lower NMSE during inference. The delay in each bank was set to 1 symbol time and feedback strength was set to 0.5.

RC random synapses realization: so as to evaluate the impact on NMSE of parameter deviations, we fixed the number of banks and filters (*N*_*F*_ = 5, *N*_*B*_ = 5) and optimized all the other parameters so as to locate the lowest NMSE for this setup. This optimized neural network was considered as ideal. Then we generated 200 RC instances where all the parameters randomly varied using a uniform distribution, with a range of +/− 10% with respect to the ideal. The parameters subject to this perturbation were: the center frequency of each MRR and the inter-MRR transmission efficiency. In addition, following^34,35^ we assumed that for the inter-MRR connections, the effective refractive index of each waveguide varies following a normal distribution with standard deviation of *Δ*_*neff*_ = 0.15 due to roughness.

### Supplementary information

Mesaritakis_PR File

Supplementary Information

## Data Availability

Simulated transmission datasets are available in (https://github.com/ksozos/ROSS_NN) (https://zenodo.org/badge/latestdoi/499526668)^61^.

## Code availability

The functions generated during the current study are available in the ROSS_NN repository (https://github.com/ksozos/ROSS_NN) (https://zenodo.org/badge/latestdoi/499526668)^61^.
