# Supplementary material for: High-speed photonic neuromorphic computing using recurrent optical spectrum slicing neural networks
Source: Commun Eng. 2022 Oct 26;1:24. doi: 10.1038/s44172-022-00024-5 (PMC10955832; doi:10.1038/s44172-022-00024-5)
Supplement: Supplementary file 2 — Supplementary Information [file 44172_2022_24_MOESM2_ESM.pdf]

High Speed Photonic Neuromorphic Computing Using Recurrent Optical Spectrum Slicing Neural Networks: Supplementary Material

K. Sozos[1], A. Bogris[1], P. Bienstman[2], G. Sarantoglou[3], S. Deligiannidis[1], C. Mesaritakis[3]

[1]Dept. of Informatics and Computer Engineering, University of West Attica, Egaleo, Greece

[2]Dept. of Information Technology, Ghent University-imec, Gent, Belgium

[3]Dept. Information and Communication Systems Engineering, Engineering School, University of the Aegean, Samos, Greece

This document provides supplementary information to "High Speed Photonic Neuromorphic Computing Using Recurrent Optical Spectrum Slicing Neural Networks", including details on the transfer function characteristics of the ROSS-NN, the optimization of ROSS-NN in high baud rate telecom applications, its memory capacity as well as a more extensive conversation about the readout layer.

Supplementary Discussion 1. ROSS-NN node transfer function characteristics

The neuromorphic node is a described as a filter-in-a-loop followed by a nonlinear element (photodiode in this paper). The recurrent filter properties define the weights of the filter in the spectral domain and its nonlinear impact as the output of the filter that will be driven to a photodetector will finally provide intensity being equal to $P_{out}=|h_{node}(t)*x(t)|^2$ where $h(t)$ is the impulse response of the filter-in-a-loop related to the transfer function of (1) (the same as the one appearing in the paper, also presented here for the sake of completeness).

$$H_{node}(f) = \frac{\sqrt{1-a}\,\sqrt{1-b}H(f)}{1+\sqrt{a\,bL}\;H(f)\,e^{-i(2\pi fT_d+\varphi)}} \quad (1)$$

$$h_{node}(t) = \mathcal{F}^{-1}[H_{node}(f)] \quad (2)$$

Interesting spectral characteristics emerge from the transfer function of the filter, such as selectivity and unequal amplitude response for frequencies being symmetrically placed with respect to central frequency. The versatile frequency response of the filter-in-a-loop provides also interesting properties as far as memory capacity is concerned as it will be shown in a subsequent paragraph. The transfer function can be programmed by adjusting critical parameters such as feedback delay time (T_d), feedback strength (depending on a, b, L parameters), the phase shift (φ) of the feedback loop and the frequency offset with respect to central frequency of the input signal. In fig.1, the transfer function of a node is plotted in different scenarios where each hyperparameter is varied. A variety of amplitude (and phase) responses can be obtained. For the sake of simplicity here we consider the transfer function of a first order Butterworth filter regarding $H(f)$. The bandwidth of the filter is set to 35 GHz. Apart from T_d which is a relatively hard parameter with minimal tuning flexibility, the other parameters can be easily tuned for an implemented chip.

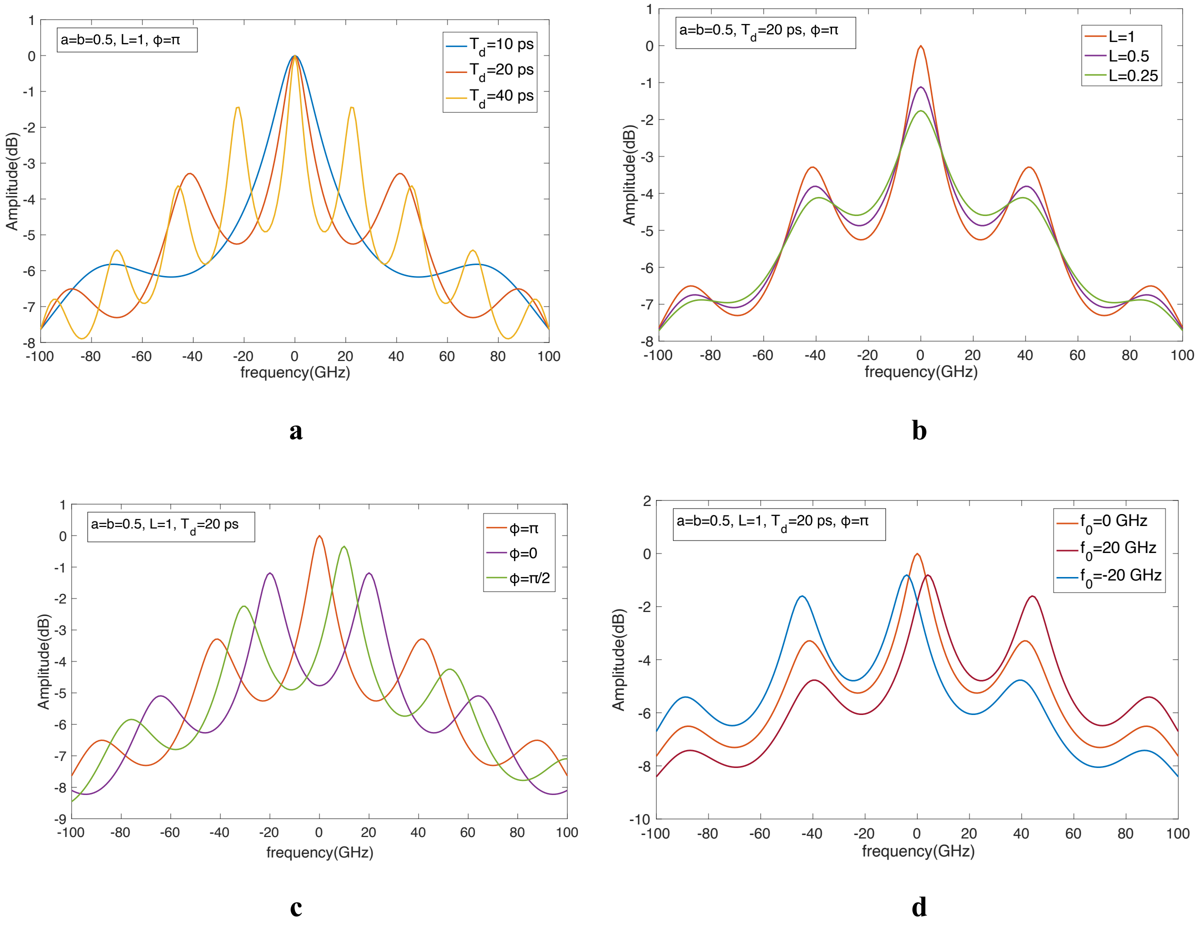

Supplementary figure 1. **Node transfer function as a function of critical parameters.** a) Effect of time delay T_d, b) effect of feedback strength L, c) effect of phase shift φ, d) effect of frequency offset f_0 with respect to signal's central frequency.

Supplementary Discussion 2. Readout Layer and hyperparameters role in the mitigation of transmission impairments

As mentioned in the manuscript, the linear readout layer after the filter nodes, could be implemented either with optical weighting and a single PD and ADC (optical readout), or with a PD and ADC pair after each node followed by a digital linear regression unit (digital readout). The two main approaches are depicted below.

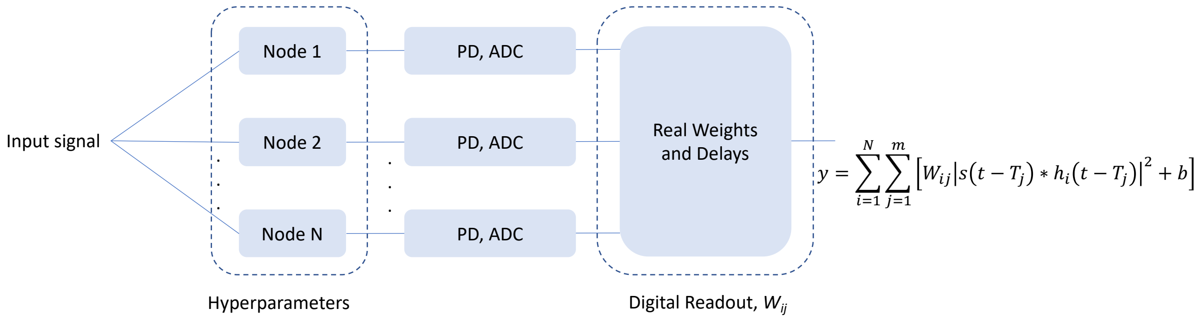

Supplementary figure 2. **Digital readout for real signals (PAM-4, NARMA)**

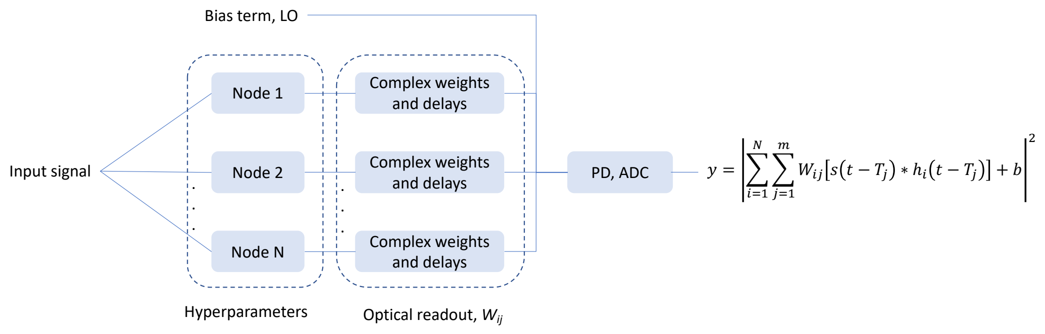

Supplementary figure 3. **Optical readout**

In the manuscript, we present results that rely on electrical readout depicted in fig. 2 which is more straightforward to implement in real-life. That is, the output of each recurrent filter is photodetected, sampled and then sent to a linear regression stage where we have also included memory at the resolution of a symbol so as to correlate past and future symbols with respect to the current one as a typical Feed Forward Equalizer (FFE) does. Therefore, if we have to include k delays taps in the FFE, where k=2m+1, then the symbols s_{i-m} to s_{i+m} will be considered as inputs in the linear regression part per filter output. This approach is followed in dispersion mitigation problems presented for PAM-4, 16-QAM in the paper. The number of delay taps k depends on the memory of fiber channel which is proportional to the group delay time T=D*$\Delta\lambda$*L_D, where D is the second order dispersion parameter, $\Delta\lambda$ the optical bandwidth occupied by the signal and L_D the transmission distance. In NARMA-10 benchmark we use only 10 taps for past symbols in the readout.

In figure 3, we depict optical regression based on [1]. This approach requires less opto-electronic and electronic components (PD, ADC) and as shown in the equations included in fig. 3, it enhances the coherent interaction of the spectral components coming from all nodes resulting in more complex nonlinear activation after photodetection. This is also reflected on the BER results, not shown in a graph, which present a significant improvement, exceeding an order of magnitude, compared to the ones presented in fig. 3 of the manuscript. However, optical readout requires coherent interaction with an optical bias which is equivalent to implementing coherent detection that is sensitive to phase noise and frequency fluctuations. In that case, extra DSP is required in order to attain carrier synchronization. Moreover, the training procedure of the optical weighting unit may be challenging, as extra delay lines equipped with phase shifters and variable optical attenuators are required. All the aforementioned difficulties are avoided taking advantage of digital readout using a few (two or three in high baud rate telecom systems) lower bandwidth receivers which reduce the necessity for high bandwidth operation for both PDs and ADCs.

When coherent signals are sent to the digital readout, then two readout units are trained, one per quadrature as depicted in fig. 4.

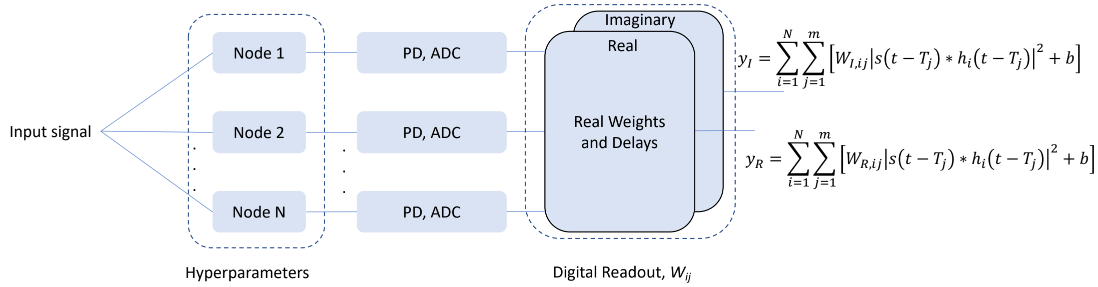

Supplementary figure 4. **Digital readout for complex signals (QAM-4)**

In PAM-4 and QAM-16 transmission systems presented in the manuscript we perform a complete scanning of critical hyperparameters of each node, as the ones presented in fig. 1 of supplementary information, in order to identify regimes of better equalization performance. For instance, the desired diversity in Chromatic Dispersion (CD) mitigation was achieved through the versatile transfer function of the recurrent node which depends on the hyperparameters of filter bandwidth, frequency offset with respect to the central frequency of the incoming signal. Regarding the rest parameters, L=1, a=b=0.5 and $\varphi=\pi$, $T_d=9$ *ps*. In fig.5, a cross investigation of the two important parameters, filter bandwidth and frequency offset, is carried out, illustrating the conditions under which the best differentiation and optimum performance is achieved when CD is the dominant impairment in 112 Gbaud PAM-4 system. A comparison is carried out between receivers using filters with and without feedback, showing the superiority of recurrent processing (as shown in left and right subfigures of fig. 5). More specifically, in the presence of feedback loop, the system transforms to a high-dimensional dynamical system and this is also evident in the BER performance (an order of magnitude improvement). These results were achieved considering simple Mach-Zehnder Delay Interferometers (MZDI) filters in the loop. In a real system, the bandwidth of the recurrent filter and the delay are hard parameters, not easy to tune. However, for a different bandwidth or delay parameter, there is a flexibility in acquiring very good performance if frequency offset is properly tuned as shown in fig. 5.

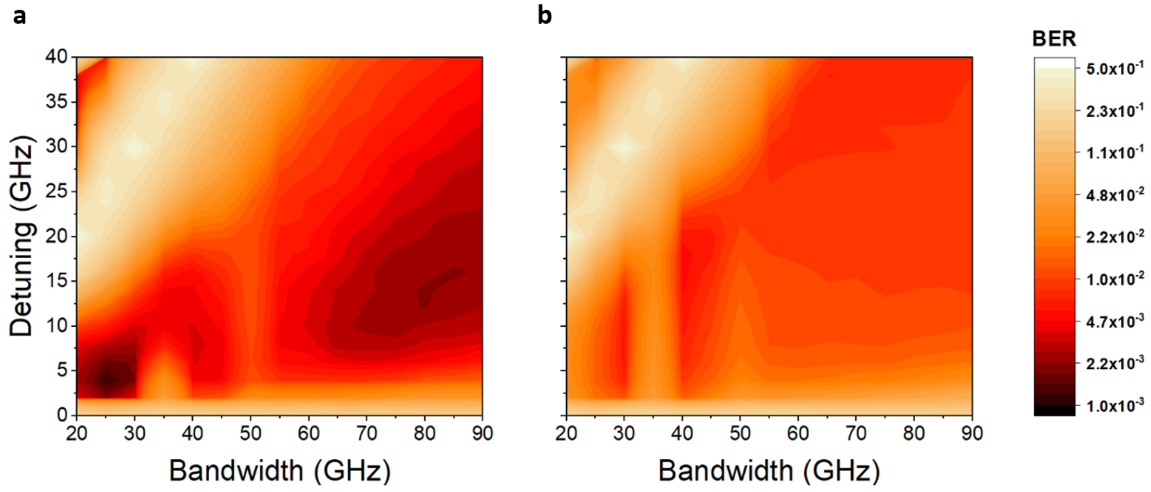

Supplementary figure 5. **BER performance as a function of filter bandwidth and frequency detuning in respect to the carrier.** The benchmark is numerically carried out considering a 40 km O-band link with a GVD parameter D=3 ps/nm km. a) The proposed RC system with two recurrent nodes. The two filters are partially overlapping with acceptable results in a wide bandwidth area. For small filter bandwidth, a frequency detuning of 5 GHz is enough for good differentiation, while the greater the bandwidth the greater the flexibility with respect to inter-filter detuning. 6) The system of two simple filters providing frequency diversity shows inferior results with a similar trend. The periodicity that is observed in the BER results is due to the periodical spectrum of the MZDI.

Regarding QAM-16 detection, our approach is an alternative to KK approach [2] that can extract both quadratures with the use of direct detection. Our scheme takes advantage of the strong residual carrier and recurrent filtering so as to differentiate the 16 or 32 symbols at the output. If one uses simple filters, it is not possible to extract the symbols efficiently, therefore the recurrent operation is indispensable in this example as well. The coherent transmission is performed in O-band with D=3 ps/nm*km and the optimization of the system is carried out based on filter frequency and inter-filter detuning (contour plot of fig. 6b). The rest of the parameters, such as the feedback Delay Time (T_d), Feedback Strength (L) and the Carrier-to-Signal-Power-Ratio are also optimized in extensive simulation trials. The role of the transmitter linewidth is also investigated and the results are presented in the fig. 6a. The relation between BER results and linewidth reveals that the proposed receiver is very tolerant to transmitter phase noise. BER degrades moderately (from $4\text{x}10^{-3}$ to $9\text{x}10^{-3}$) when linewidth increases from 1 kHz to 200 kHz whilst typical coherent receivers demand the use of narrow linewidth lasers (< 30 kHz). Fig. 7 presents indicative scatterplots. Fig. 7a shows the constellation of the transmitted signal in the presence of a strong residual carrier which

packages all QAM-16 symbols to the first quadrant. Fig. 7b shows the output constellation when filters without feedback are used. It becomes evident that it is not easy for the linear equalizer of the digital readout to classify the 16 symbols, which are characterized by strong nonlinearities in the constellation diagram. On the contrary, when recurrent filters are utilized (fig. 7c), the classification performance vastly improves using the same readout and the BER becomes equal to $5x10^{-3}$.

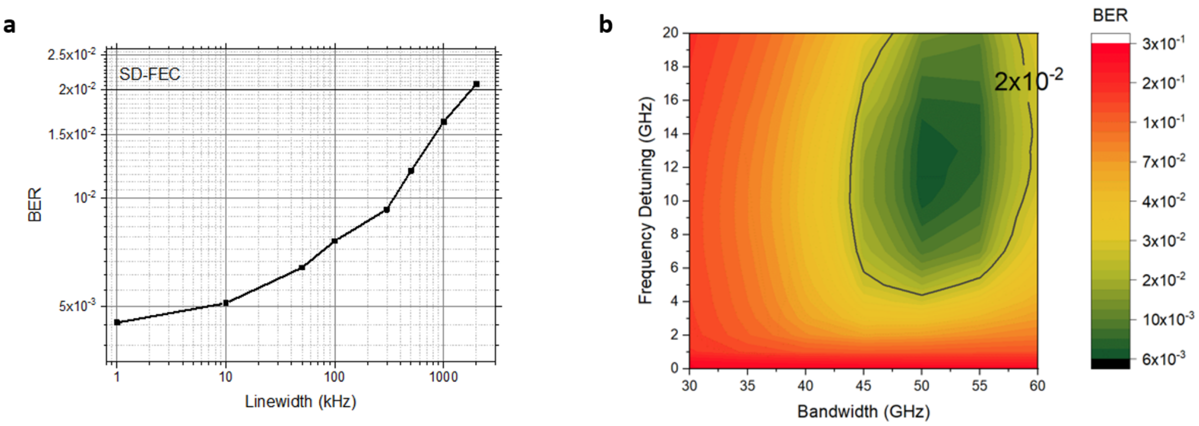

Supplementary figure 6. The dependence of the coherent system's performance on transmitter linewidth and on the receiver parameters. a) The BER performance is not severely affected by an exponential increase in the transmitter linewidth. This is attributed to the direct detection nature of the receiver. b) The contour plot of BER performance as a function of bandwidth, detuning for the 3 recurrent nodes with L=0.5, T_d=9 ps of the receiver. The first filter is centered on the carrier frequency while the other two are detuned symmetrically in the blueshifted and redshifted frequency components. The best results are achieved for 50 GHz bandwidth proving that spectral slicing relaxes the need for high bandwidth photodetectors and ADCs.

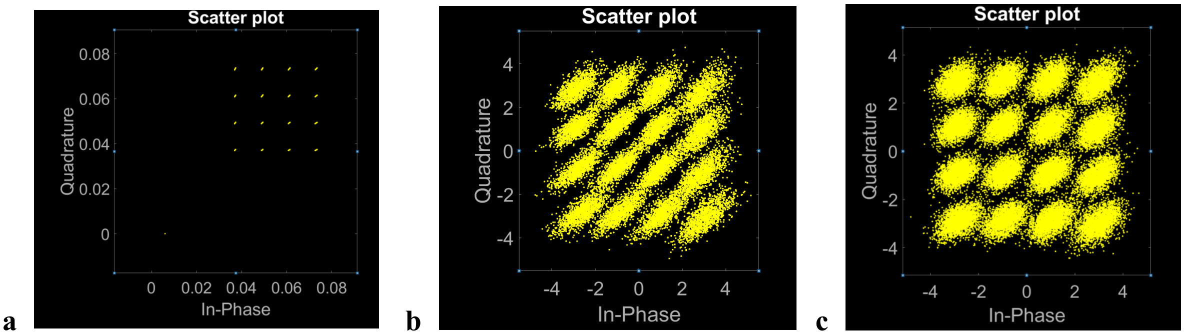

Supplementary figure 7. Scatterplots of the transmitted and received QAM-16 signal a) The transmitted QAM-16 signal with an increased residual carrier following the paradigm of self-coherent techniques b) The received signal after 20 km of O-band transmission with D=3 ps/nm/km and after being processed by 3 simple filters. The BER performance is in the order of $3x10^{-2}$. c) The signal received and processed by 3 recurrent nodes with BW=50 GHz, Frequency detuning=15 GHz, L=0.5, T_d=9 ps, achieving BER performance near the HD-FEC limit ($5x10^{-3}$).

Supplementary Discussion 3. Memory Capacity

As already reported, the recurrent nature of the optical filter is anticipated to increase its fading memory, which is required in problems such as dispersion mitigation. In order to estimate the short-term memory of the recurrent node, we solve the memory capacity task. Memory capacity MC is derived from the memory

function $m(i)$ of eq. (3) [3], [4] and quantifies the amount of information from past symbols that a recurrent system can reproduce.

$$m(i) = \frac{(y(n-1)\ o_i(n))^2}{\sigma^2(\ y(n))\ \sigma^2(o_i(n))} \tag{3}$$

In (3), $y(n)$ is a random input signal in the range [0, 1], $o_i(n)$ is system output at time n when the output weights are trained to reproduce the i-th past input signal $y(n-i)$. σ^2 is the variance. Then, the memory capacity MC is defined as the sum of $m(i)$ as follows:

$$MC = \int_1^\infty m(i) \tag{4}$$

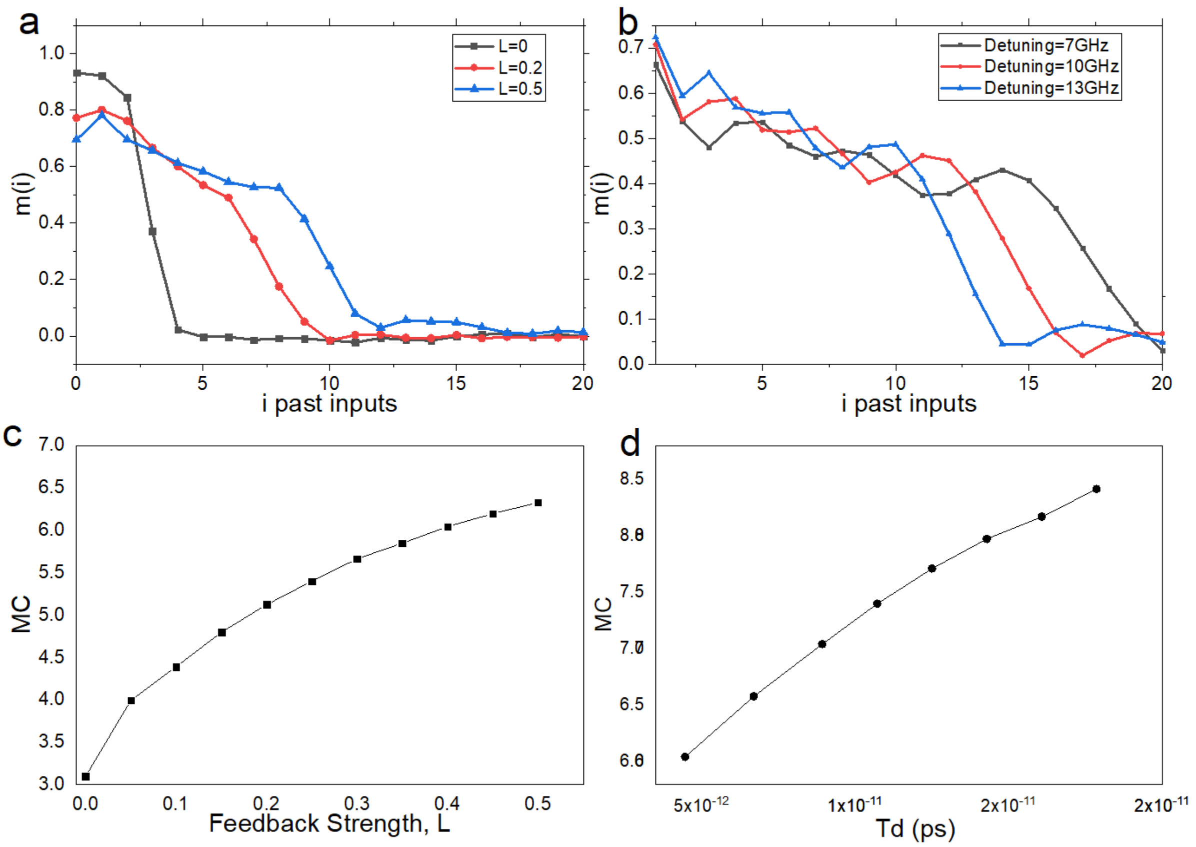

Supplementary figure 8. Memory capacity studies. a) Memory function $m(i)$ in respect of different feedback strength values L and b) of different frequency detuning values between the filters. c) The memory capacity of a system with four parallel recurrent nodes as a function of feedback strength L, and d) as a function of Time Delay of the feedback loop T_d. Tuning the two feedback parameters the memory ranges from 3 to 8.5 while the memory functions can spread even across 20 symbols in the past.

We calculate memory capacity for ROSS-NN considering N_B=4 and N_F=1, that is four recurrent nodes that slice input signal in four bands. We investigate the effect of feedback strength L, along with other important

parameters such as filter bandwidth and frequency detuning. In fig. 8a we observe that *m* increases when feedback strength is increased from zero to 50%. The fact that even without feedback a good correlation is achieved for one or two symbols is attributed to the intrinsic memory of the filter. Another important result arises when the frequency detuning between the nodes is tuned (fig. 8b). We consider four filters with detuning between the *i*-th and the *j*-th filter defined as $\Delta f_{ij}=(j-i)\Delta f$ with Δf taking three values (7 GHz, 10 GHz, 13 GHz). All filters have 40 GHz 3dB bandwidth and the first filter is tuned to the central frequency of the incoming random signal. As Δf scales from 7 to 13 GHz, we observe that fading memory extends to less symbols in the past. Therefore frequency detuning between different nodes also tunes the fading memory of the system. Fig. 8c calculates MC as a function of feedback strength. It is obvious that as feedback increases, the system is capable of holding information of more symbols from the past. The same trend is observed with the increase of feedback delay time (fig. 8d). Memory capacity scales as a square root function for the specific values of feedback delay time and frequency detuning. This square root behavior is observed in similar works [5].

Supplementary References

1. M. Freiberger, A. Katumba, P. Bienstman and J. Dambre. Training Passive Photonic Reservoirs with Integrated Optical Readout. Transactions on Neural Networks and Learning Systems **30**, 1943-1953 (2019)
2. D. Zou, Q. Zhang, W. Wang, Q. Sui, X. Yi, Z. Li, X. Gong, and F. L. 100 GBaud SSB PAM-4 Signal Transmission over 80-km SMF with THP and KK Receiver. Optical Fiber Communication Conference, (2021)
3. Jaeger, H. Short term memory in echo state networks. Tech. rep. GMD report 152. German National Research Center for Information Technology (2001)
4. Michiel Hermans, Benjamin Schrauwen. Memory in linear recurrent neural networks in continuous time. Neural Networks **23**, 341-355 (2010)
5. C. Sugano, K. Kanno and A. Uchida. Reservoir Computing Using Multiple Lasers With Feedback on a Photonic Integrated Circuit. Journal of Selected Topics in Quantum Electronics **26**, 1-9 (2020)
