# Supplementary material for: High-speed photonic neuromorphic computing using recurrent optical spectrum slicing neural networks
Source: Commun Eng. 2022 Oct 26;1:24. doi: 10.1038/s44172-022-00024-5 (PMC10955832; doi:10.1038/s44172-022-00024-5)
Supplement: Supplementary file 1 — Mesaritakis_PR File [file 44172_2022_24_MOESM1_ESM.pdf]

Peer Review File

High-Speed Photonic Neuromorphic Computing Using Recurrent Optical Spectrum Slicing Neural Networks

Open Access This file is licensed under a Creative Commons Attribution 4.0 International License, which permits use, sharing, adaptation, distribution and reproduction in any medium or format, as long as you give appropriate credit to the original author(s) and the source, provide a link to the Creative Commons license, and indicate if changes were made. In the cases where the authors are anonymous, such as is the case for the reports of anonymous peer reviewers, author attribution should be to 'Anonymous Referee' followed by a clear attribution to the source work. The images or other third party material in this file are included in the article's Creative Commons license, unless indicated otherwise in a credit line to the material. If material is not included in the article's Creative Commons license and your intended use is not permitted by statutory regulation or exceeds the permitted use, you will need to obtain permission directly from the copyright holder. To view a copy of this license, visit http://creativecommons.org/licenses/by/4.0/.

Reviewers' comments:

Reviewer #1 (Remarks to the Author):

In “High-Speed Photonic Neuromorphic Computing Using Recurrent Optical Spectrum Slicing Neural Networks,” Sozos et al. discuss and numerically investigate an array of spectral filters combined with temporal delays for unconventional photonic information processing. In their concept, an optically encoded signal is ‘spliced’ into various copies, which are then (i) linearly transformed in a long roundtrip time resonator that also (ii) includes a spectral filter with a tunable central frequency and filter bandwidth. The output of these filter bands are than detected, and the combined signals are used to solved computational benchmark test such as the NARMA tasks as well as the reconditioning of high-bandwidth optical communication channels. The numerical results appear to be sound, and in principle the conclusions drawn from the data are relevant and reliable.
At the moment I cannot recommend publication essentially because I feel that the work does not provide a fair comparison to the relevant and previous work in the field.
First and foremost, I feel that several crucial citations are not implemented. The authors otherwise provide a well balanced intro to the field (besides maybe be the citation of spatially distributed nodes, where the first demonstration of a fully implemented system was done by Porte, et al., Journal of Physics: Photonics 2021).

First, the paper “Machine-learning-based equalization for short-reach transmission: neural networks and reservoir computing” by Da Ros (https://doi.org/10.1117/12.2583011) even introduced the concept of spectral slicing, and for me therefore has to be referenced.

Second, the reconditioning of optical communication channels based on photonic neural network implementations was pioneered, numerically and in experiments, by Argyris, who has written multiple papers on the topic including a review. A sound introduction to the field therefore needs to reference the work of this author.

Finally, I find the authors overstate the quality of their results at several places. For example they claim “remarkable results” or comparable numerous times, which I personally find over-selling. What, however, is questionable is that this is done in comparison between their work and reference [28] when applied to the NARMA10 task. The problem is that the authors here only report numerical results, while reference [28] is experimental. In particular in the context of the NARMA10 task, all the different possible noise sources are highly relevant, and hence this is not a fair comparison and the fact that numerics are compared to experimental implementations should be more clearly states.

Minor comments:
Typo: “loop with delay T_{d,,.}” ->“loop with delay T_{d}.”
Typo: “In an RC-like treatment” -> “In a RC-like treatment”

Reviewer #2 (Remarks to the Author):

High-Speed Photonic Neuromorphic Computing Using Recurrent Optical Spectrum Slicing Neural Networks
Sozos et al.

This paper proposes a reservoir computer where the reservoir is implemented using a fiber loop and the reservoir's nonlinearity is provided by spectral filters. The authors use numerical simulations to evaluate the performance of their proposed system, and demonstrate its ability with a canonical machine-learning task (NARMA10 timeseries prediction) as well as some more specialized tasks related to optical communications. The latter especially make sense as the input to the reservoir is then natively optical.

Based on my admittedly incomplete knowledge of the prior work in time-loop photonic reservoir computers, this paper's proposal is novel and the numerical results suggest promise. I believe it is a solid contribution to the literature on photonic reservoir computers and I recommend it for publication in Communications Engineering, subject to the authors responding to the revisions suggested below.

More detailed remarks:

The abstract begins with "achieving machine learning processing at the picosecond scale, with minimum power consumption". What timescale did this proposal achieve (in numerical simulations)? What power consumption did it achieve? I suggest providing both the projected power consumption for the optical part of the system and for the electronic part of the system (given separately, so that the reader can easily tell how much was optical and how much was electronic).

The abstract says, "The performance enhancement surpasses state-of-theart digital processing techniques by doubling the reach while minimizing complexity and power consumption by a factor of 10 compared to state-of-the-art solutions." If the word count allows, it would be nice for the abstract to mention concretely a.) what the state-of-the-art is; b.) what performance the state-of-the-art achieves; c.) what performance the proposed solution is predicted to achieve.

I recommend the authors cite "Scalable reservoir computing on coherent linear photonic processor" by Nakajima et al. (2021). This work experimentally demonstrates a photonic reservoir computer based on photonic loops and filtering, with high-bandwidth input signals, so has a fairly significant overlap with Sozos et al.'s proposal.

The authors should make the code and data associated with this paper available in a public repository. I was not able to find the code for the simulations reported in the paper and this is a major shortcoming regarding reproducibility.

There are a number of typos in the paper. I would recommend a thorough proof-reading to eliminate these. For example:
"In this paper we have proposed and numerically evaluated a new neuromorphic photonic concept based on recurrent optical spectrum slicing; implemented by optical filters embedded in a delay loop." -- I don't think the semicolon should be there (or if one would like the pause, then I think an extra joining word is needed.

"optical filters placed in an loop" should be "optical filters placed in a loop"

Reviewer #3 (Remarks to the Author):

The authors report on a machine learning algorithm that can be implemented in a photonic format, making it potentially able to perform real-time processing of optical signals.

While the manuscript is interesting, it has several shortcomings that need to be addressed before it can be recommended for publication.

1) The authors imply that the filtering performs a nonlinear transformation of the input signal. This is not the case and needs to be carefully discussed in the text. Any passive linear optical system is linear whether described in the frequency domain or the time domain and they are just related by a Fourier transform. The only nonlinear in the system is due to the detectors and this needs to be stated.

2) Considering point 1), there is really no difference between this work and Katumba et al., Sci. Rep. 8, 2653 (2018) or Vandoorne et al., Nat. Comm. 5, 3541 (2018). The microring resonators used in

these studies also act as spectral filters with random resonance frequencies. While the implementation might be simpler in the present manuscript, the underlying concepts appear to be the same.

3) I might have missed it, but the authors do not appear to state the true processing speed. In their implementation, they need to apply the trained weights to the measured signal. This is always the bottleneck in any physical reservoir. They say that they have a fast processor to do this but they never give the speed that it can operate. They need to give this detail and they need to state the true throughput of their system <including> applying the weights.

4) They do not explain <why> their system is equivalent to a reservoir computer. The filtering is equivalent, in the time domain, to performing a convolution of the filter functon(in the time domain) with the incoming signal (in the time domain). There are several works that describe how convolutions, via a Volterra series expansion, can be a way to represent any function. The authors should consult this older reference: Boyd et al, IEEE Trans. Circuits Sys. CAS-32, 1150 (1985) and more recent works such as Gonon et al., IEEE Trans. Neural Netw. Learn. Syst. 31, 100 (2020), which should help them explain why their method works.

5) The idea of spectral filtering of signals to improve the performance of an RC has already been given in Carroll, Physica D 416, 132798 (2021). The authors need to put their work in the context of this previous work.

Overall, I see very little improvement in our understanding of machine learning in this manuscript - they appear to be repackaging what is already known in the literature and they don't give a compelling explanation as to why their method works. So the main possible reason for publication is that they truly have a high-throughput system. But they do not present compelling evidence that they have done so unless they can demonstrate fast full-system throughput including applying the weights.

5) The error rates on the tasks are fairly high. The tasks are simple enough that a linear classifier can do a reasonable job. To show that their method does better than a linear classifier, which is a minimum baseline, the authors need to compare their error rates to a linear classifier.

Paper Title: High-Speed Photonic Neuromorphic Computing Using Recurrent Optical Spectrum Slicing Neural Networks

Response to reviewers

The authors would like to thank all the reviewers for their valuable comments. We have taken into account all reviewers' suggestions and by revising the manuscript we believe that its quality has been significantly improved. The response per comment of each reviewer is provided below.

Reviewer #1 (Remarks to the Author)

General comment: In "High-Speed Photonic Neuromorphic Computing Using Recurrent Optical Spectrum Slicing Neural Networks," Sozos et al. discuss and numerically investigate an array of spectral filters combined with temporal delays for unconventional photonic information processing. In their concept, an optically encoded signal is 'spliced' into various copies, which are then (i) linearly transformed in a long roundtrip time resonator that also (ii) includes a spectral filter with a tunable central frequency and filter bandwidth. The output of these filter bands are than detected, and the combined signals are used to solved computational benchmark test such as the NARMA tasks as well as the reconditioning of high-bandwidth optical communication channels. The numerical results appear to be sound, and in principle the conclusions drawn from the data are relevant and reliable.

At the moment I cannot recommend publication essentially because I feel that the work does not provide a fair comparison to the relevant and previous work in the field.

Response to general comment: We would like to thank the reviewer for his/her positive comments regarding the technical soundness of our work. We have addressed the skepticism related to fair comparison to the relevant and previous work in the field in the individual comments that follow.

Reviewer's comment 1

First and foremost, I feel that several crucial citations are not implemented. The authors otherwise provide a well-balanced intro to the field (besides maybe be the citation of spatially distributed nodes, where the first demonstration of a fully implemented system was done by Porte, et al., Journal of Physics: Photonics 2021). First, the paper "Machine-learning-based equalization for short-reach transmission: neural networks and reservoir computing" by Da Ros (https://doi.org/10.1117/12.2583011) even introduced the concept of spectral slicing, and for me therefore has to be referenced. Second, the reconditioning of optical communication channels based on photonic neural network implementations was pioneered, numerically and in experiments, by Argyris, who has written multiple papers on the topic including a review. A sound introduction to the field therefore needs to reference the work of this author.

Authors' response

We would like to thank the reviewer for bringing to our attention Porte et. al. work. We will include it in the references list.

The work of Da Ross et. al., is very relevant to our work, for this reason it was already cited in the original manuscript very clearly. In specific, in page 3, left column we have written: *"Spectral slicing, through simple non-recurrent filters, before RC processing was first proposed in [22] where the output of each filter was sent to a digitally implemented reservoir computing network",* where [ref. 22] in the original manuscript is *"S. M. Ranzini, R. Dischler, F. Da Ros, H. Bülow and D. Zibar. Experimental Investigation of Optoelectronic Receiver With Reservoir Computing in Short Reach Optical Fiber Communications. Journal of Lightwave Technology, 39, 2460-2467 (2021)"]* which presents experimental results of the idea based on signal filtering, through typical bandpass filters and the use of a reservoir computing at the digital domain. For the sake of completeness, in the revised manuscript we also included "Machine-learning-based equalization for short-reach transmission: neural networks and reservoir computing" by Da Ros (https://doi.org/10.1117/12.2583011) proposed by the reviewer. In the original manuscript, we also highlight the main difference of our work to that of Da Ros and his co-workers, which is to bring the recurrent processing in the optical domain so as to reduce the DSP footprint of the overall device. In order to better highlight the difference of our approach with Da Ros' ideas we have added a part in the revised manuscript so as to better highlight the different approach followed in this work (page 2, right column in the revised manuscript).

Following the reviewer's suggestion we also included relevant works of A. Argyris et.al. We agree that although Argyris' TDRC approach for signal equalization has critical shortcomings when real-time implementation at very high baud rates is required, it is one of the key works using RC for telecom applications and therefore an introduction should definitely include it. Based on this fact, we moved a step further; we compare the numerical results of transmission performance of our approach versus the simulation results presented by all the relevant works (including Argyris'), utilizing photonic RC schemes for optical communications, in terms of the well-established metric of bitrate*distance product. Table I in the revised manuscript clearly demonstrates the advantages of the ROSS-NN approach over state of the art approaches in terms of both bitrate performance and its need for only one sample per symbol in order to provide dispersion equalization.

Reviewer's comment 2

Finally, I find the authors overstate the quality of their results at several places. For example they claim "remarkable results" or comparable numerous times, which I personally find over-selling. What, however, is questionable is that this is done in comparison between their work and reference [28] when applied to the NARMA10 task. The problem is that the authors here only report numerical results, while reference [28] is experimental. In particular in the context of the NARMA10 task, all the different possible noise sources are highly relevant, and hence this is not a fair comparison and the fact that numerics are compared to experimental implementations should be more clearly states.

Authors' response

Regarding the reviewer's comment about overselling the proposed work, it is our strong belief that ROSS-NN has some unique properties related to its ability to handle ultra-broad optical signals (> THz bandwidth) through spectral slicing and optical recurrent processing. Its merits really shine when ultra-

fast, inherently optical, data are processed. Especially, in the key area of high-speed short reach optical communication systems, the need of industry is to extend the reach beyond 10 km at 100 Gbaud (800 Gb Ethernet technologies is the driver) in order to serve inter-DCN communication scenarios [Nagarajan, R., Lyubomirsky, I., Jan **13**, 2021 COBO Webcast, Inphi Moves Big Data Faster Next-Gen Data Center Interconnects: The Race to 800G]. The only viable solution to accomplish this target is coherent technology, which, however, is prohibitive in terms of both power consumption and digital signal processing capabilities required. Our work provides an alternative route to achieve this goal with much lower power consumption (we included a detailed comment in the section of the optical communication task in page 6, left column of the revised manuscript that showcases a multi-watt reduction with the use of ROSS-NN) and DSP complexity (only FFE is required at the back-end).

Thus, a combination of superior BER performance along with simplicity and power efficiency makes our proposition unique at high baud rate optical post processing. These statements are validated by the results shown in Figure 3b and in Table I. In particular, the manuscript is indicative of the superior BER performance of ROSS-NN over the more complex MLSE, VNLE sophisticated DSP techniques and as mentioned above versus all state-of-the-art RC based implementations. Nonetheless, in order to follow the reviewer's suggestion, we have modified specific sentences in the revised manuscript so as to remove any suspicion of over-selling.

Regarding the second part of the reviewer's comment related to NARMA 10 benchmarks, it is true that our work is purely numerical. However, our simulations take into account all sources of noise at the detectors (thermal, shot, etc) and simulate typical microring resonators as optical filters in terms of radius, losses and coupling coefficients. In this respect, we believe that our results should not overestimate the performance of a real-life ROSS-NN implementation. However, we removed the comparison with the experimental investigation of the reference [28] and replaced it with another, demonstrating numerical, results from similar works for a fair comparison (see changes in page 5 and references 14, 36 and 37 of the revised manuscript). Finally, an issue raised by the reviewer, is performance in terms of the NARMA 10 task. As state above, the main performance enhancement of our scheme compared to the state of the art is clearly depicted and confirmed when IM/DD equalization is taken into consideration. This is validated by benchmarking ROSS-NN versus multiple approaches (neuromorphic included) and by comparing simulation results. The NARMA 10 task has been chosen in the first part of our result section, so as to clarify that ROSS-NN is not a task dependent model that can address specific applications but, on the contrary, it can be considered a generic recurrent/reservoir computing implementation. In this context, we demonstrated results that are similar to the state of the art.

Furthermore, the most critical part, that although present in the original manuscript, it is not highlighted properly, is that between the NARMA and the IM/DD task there is a critical difference that is the basis for the performance boost. In the case of NARMA digital samples are used to amplitude modulate an ideal optical carrier and then are injected into the ROSS-NN. Therefore, the input signal is not inherently optical, but it is transferred in the optical domain. Based on this, the input signal does not contain any phase information. On the other hand, in the IM/DD case, the input signal occurs through the accurate simulation of SMF propagation and the signal that is computed is complex, thus it contains useful information in the phase domain which is leveraged by ROSS-NN in order to mitigate dispersion effects. A discussion has been included in the last paragraph of NARMA section in order to highlight this difference.

Reviewer's comment 3

Minor comments:

Typo: “loop with delay T_{d,,.}” ->“loop with delay T_{d}.”

Typo: “In an RC-like treatment” -> “In a RC-like treatment”

Authors’ response

We would like to thank the reviewer for carefully reading the manuscript. We have corrected the typos.

Reviewer #2 (Remarks to the Author)

High-Speed Photonic Neuromorphic Computing Using Recurrent Optical Spectrum Slicing Neural Networks Sozos et al.

General comment: This paper proposes a reservoir computer where the reservoir is implemented using a fiber loop and the reservoir's nonlinearity is provided by spectral filters. The authors use numerical simulations to evaluate the performance of their proposed system, and demonstrate its ability with a canonical machine-learning task (NARMA10 timeseries prediction) as well as some more specialized tasks related to optical communications. The latter especially make sense as the input to the reservoir is then natively optical.

Based on my admittedly incomplete knowledge of the prior work in time-loop photonic reservoir computers, this paper's proposal is novel and the numerical results suggest promise. I believe it is a solid contribution to the literature on photonic reservoir computers and I recommend it for publication in Communications Engineering, subject to the authors responding to the revisions suggested below.

Response to general comment: We would like to thank the reviewer for his/her positive comments.

More detailed remarks:

Reviewer’s comment 1

The abstract begins with "achieving machine learning processing at the picosecond scale, with minimum power consumption". What timescale did this proposal achieve (in numerical simulations)? What power consumption did it achieve? I suggest providing both the projected power consumption for the optical part of the system and for the electronic part of the system (given separately, so that the reader can easily tell how much was optical and how much was electronic).

Authors’ response

This is an extremely important aspect of such systems and a good remark. The timescale of our system is limited only by the electro-optic conversions in the output layer, as we have mentioned in the manuscript. With the state-of-the-art Analog-to-Digital converters in our mind, which approach 200 GHz of sampling frequency, we envisage responses in the picosecond scale. This is in contrast to the time-delayed RC works in literature, where a masking process is a prerequisite, and the processing speed is a fraction of this number (usually 8 to 24 times smaller for acquiring the corresponding number of virtual nodes). In the present work we simulated a system up to 112 GBaud which corresponds to ~9 ps timescale. In order to address reviewer's point, we included in the introduction the following text: *"The simulated system achieves real-time processing with almost zero latency at tasks exceeding 100 Gbaud. In this work, numerical simulations show that ROSS-NN can operate at timescales of a few picoseconds."*

The power consumption of the photonic part includes only the power consumed by the phase shifters (usually in the mW regime). More importantly if non-volatile Pockel effect phase shifters are considered [Ortmann et.al. (2019), https://doi.org/10.1021/acsphotonics.9b00558] as the prime ROSS-NN tuning mechanism, power consumption will be driven to the nWatt regime, rendering the ROSS-NN scheme extremely appealing. The electronic part of the system includes the analog to digital conversion and the linear weights in the form of a FIR filter (all together in the sub-Watt regime even for the ultrafast telecom applications). We included the following comment in the 6th page, left column, for the most interesting, in terms of consumption, telecom problem.

"It must be stressed that in the strict environment of short-reach communications, the power consumption is of paramount importance. With a two-node ROSS-NN, we launch a receiver that, apart from its almost passive optical part (sub-μW consumption for state of the art phase shifters [40]), consumes less than 1 W for 112 GBaud, based on the latest 7 nm FinFET technology [41,42] for the two relaxed 40 GHz ADCs and a 50-tap FFE. For comparison, a minimal 2-tap MLSE with 128 multiplications, with a 56 GHz ADC, would consume over 1.5 W. In a transceiver with four coarse wavelength division multiplexed channels, this corresponds to more than 2 W or 20% reduction in the transceiver power envelope."

Reviewer's comment 2

The abstract says, "The performance enhancement surpasses state-of-the art digital processing techniques by doubling the reach while minimizing complexity and power consumption by a factor of 10 compared to state-of-the-art solutions." If the word count allows, it would be nice for the abstract to mention concretely a.) what the state-of-the-art is; b.) what performance the state-of-the-art achieves; c.) what performance the proposed solution is predicted to achieve.

Authors' response

The reviewer's comment is to the point, as ROSS-NN is a proposition offering low complexity – and high performance. In the original manuscript, we had provided a comparison between ROSS-NN and state-of-the-art algorithms for CD induced power fading mitigation in figure 3b in terms of BER performance and in the paragraph entitled: *"Few-node ROSS-NN as a photonic hardware accelerator in 100 Gbaud and*

beyond optical communication systems". We appropriately repeat part of this conversation in the abstract of the revised manuscript in order to better clarify the merits of our proposition.

More specifically, we added: "*ROSS-NN provides better performance compared to other digital or neuromorphic techniques. It extends the transmission reach to >60 km, in comparison to two state-of-the-art digital equalizers, namely a 3rd order Volterra and a 5-tap MLSE equalizer, that can barely reach up to 15 km. Furthermore, it relaxes complexity employing less than 100 multiplications/bit, offering tenfold reduction in power consumption with respect to these digital counterparts*".

Reviewer's comment 3

I recommend the authors cite "Scalable reservoir computing on coherent linear photonic processor" by Nakajima et al. (2021). This work experimentally demonstrates a photonic reservoir computer based on photonic loops and filtering, with high-bandwidth input signals, so has a fairly significant overlap with Sozos et al.'s proposal.

Authors' response

We would like to thank the reviewer for bringing this work to our attention. The work mentioned above is indeed a relevant RC work and we have included it in the introduction of the revised manuscript, where time-delayed RC systems are referred. This paper belongs to the category of time-delayed systems with the difference of introducing optical masking with the use of delaying waveguides at the input and the combination of multiple outputs at the spatial domain so as to propose a spatio-temporal RC variant (parallel time-delayed RCs at the output). In this respect, Nakajima's work is different to ours as it implements RC using time-multiplexing and virtual nodes. Furthermore, it does not utilize spectral slicing in order to accommodate large optical signals inside the RC and to manipulate separately different frequency bands as we do.

Reviewer's comment 4

The authors should make the code and data associated with this paper available in a public repository. I was not able to find the code for the simulations reported in the paper and this is a major shortcoming regarding reproducibility.

Authors' response

The reviewer's point is valuable. The authors are in favor of open science. For this reason, we uploaded the main functions of the receiver numerical simulation in GitHub. We have also included input-output time series from the transmission system for two transmission scenarios.

https://github.com/ksozos/ROSS_NN

Reviewer's comment 5

There are a number of typos in the paper. I would recommend a thorough proof-reading to eliminate these. For example:
"In this paper we have proposed and numerically evaluated a new neuromorphic photonic concept

based on recurrent optical spectrum slicing; implemented by optical filters embedded in a delay loop." -- I don't think the semicolon should be there (or if one would like the pause, then I think an extra joining word is needed.

"optical filters placed in an loop" should be "optical filters placed in a loop"

Authors' response

We would like to thank the reviewer for carefully reading the manuscript. We have thoroughly scanned the manuscript in order to eliminate all typos.

Reviewer #3 (Remarks to the Author):

General comment: The authors report on a machine learning algorithm that can be implemented in a photonic format, making it potentially able to perform real-time processing of optical signals.

While the manuscript is interesting, it has several shortcomings that need to be addressed before it can be recommended for publication.

Response to general comment: We would like to thank the reviewer for his/her positive comments and the detailed review which contributes to the improvement of our manuscript. We have thoroughly addressed all the comments in the following paragraphs.

Reviewer's comment 1

The authors imply that the filtering performs a nonlinear transformation of the input signal. This is not the case and needs to be carefully discussed in the text. Any passive linear optical system is linear whether described in the frequency domain or the time domain and they are just related by a Fourier transform. The only nonlinear in the system is due to the detectors and this needs to be stated.

Authors' response

The reviewer's comment is accurate, when the transformation of a complex field through a linear filter is concerned. However, if we isolate the transfer characteristics of phase (frequency) to intensity or even phase (frequency) to phase then the linear filter can act as a nonlinear activation function. This has been exploited by our group in the past in [[1]] using bandpass micro-ring filters but is also valid for all types of filters. The aforementioned concept was referred in the original manuscript. In specific, in the introduction we mentioned that: *"Second, an optical filter provides a non-linear mapping of the incoming signal's phase variations to the node's intensity [20]. Hence, the coherent interaction of spectrally sliced components on the photodiode provides a complex nonlinear activation function at the output".* This claim is

demonstrated in the curves below where output intensity and phase are depicted as a function of input phase variations (frequency):

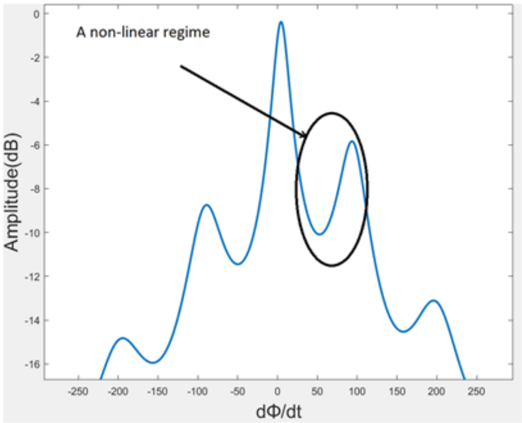

Therefore, in our work nonlinearity has a twofold origin; phase to intensity mapping and photodetection. In order to better clarify this point, we have modified the revised manuscript in page 2, left column as follows:

"Second, although an optical filter performs a linear transformation of the complex field, it also provides a non-linear mapping of the incoming signal's phase variations to the node's intensity (see supplementary information, node transfer characteristics). Hence, the coherent interaction of spectrally sliced components on the photodiode provides a complex nonlinear activation function at the output".

Reviewer's comment 2

2) Considering point 1), there is really no difference between this work and Katumba et al., Sci. Rep. 8, 2653 (2018) or Vandoorne et al., Nat. Comm. 5, 3541 (2018). The microring resonators used in these studies also act as spectral filters with random resonance frequencies. While the implementation might be simpler in the present manuscript, the underlying concepts appear to be the same.

Authors' response

It is true that our work belongs to the category of spatially developed RC systems, as the studies indicated by the reviewer. However, the mechanism assumed in both cases is intrinsically different compared to our work. On one hand, in Katumba's work the main proposition is the improvement of overall energy efficiency by replacing single-mode Y-junction waveguides with multimode versions so as to enhance the number of the observables in the system (the number of nodes) and in turn invoke a more complex dynamical behaviour. Similarly, in Vandoorne work the RC consists of passive waveguides in a mesh topology which includes feedback loops in order to attain fading memory, while the complexity triggering mechanism in this case is the coherent interaction of two signal that have accumulated a "complicated" phase shift.

To our understanding, no microring resonators are used in the aforementioned works, whereas more importantly no filtering process takes place (spectral decomposition). However, even if MRRs are introduced in the RC networks (as in [ref. 17] of the manuscript and in [[2]]), they are employed to perform only a random coherent mixing of the signal, or they are used in order to offer Kerr-related nonlinearities.

For example, the frequency detuning used in [2] serves only as relaxation of the strong absorption that the notch filters exhibit in their resonance frequency. For this reason, the detuning has the same value for all MRRs, thus neither spectral slicing nor frequency diversity from the different nodes takes place. This is evident from the following paragraph taken from the paper:

"In this figure, we have considered that the microrings are strictly the same, meaning that all 16 nonlinear microring resonators have the same resonance frequency. For more realistic simulations, we give in Fig. 6(b) - and for the same input conditions - the performance of the MR-reservoir when the resonance frequencies of the microrings are different. The resonance frequencies of the 16 microring resonators follow a Gaussian distribution centred on respectively $\delta\lambda \in \{-50, 0.0, 50, 100\}$ pm, with a 10 pm standard deviation, that is a rather pessimistic value with regard to the current technology."

This explains that any resonance mismatch is considered here only as an imperfection and lies in the order of few hundred MHz on the "pessimistic" scenario.

Therefore, the aforementioned works do not perform any kind of spectral slicing, they instead handle the entire input spectrum as a whole. The only work that introduces spectral slicing is ref. [22] of the original manuscript which however relies on the use of a rather heavy DSP in the form of software RC in order to perform nonlinear processing. On the contrary, our work introduces recurrent spectrum slicing in the optical domain and its unique properties (diversity in the frequency domain, enhanced nonlinear behavior), which we believe is a substantial difference with respect to Katumba's and Vandoorne's works and other relevant works in the literature as the ones presented above.

In order to clarify this point, we have added in the revised manuscript, in the second-to-last paragraph of the introduction that: *"All the aforementioned works do not rely on any type of spectral slicing and optical processing of selected frequency components in the optical domain."*

Reviewer's comment 3

3) I might have missed it, but the authors do not appear to state the true processing speed. In their implementation, they need to apply the trained weights to the measured signal. This is always the bottleneck in any physical reservoir. They say that they have a fast processor to do this but they never give the speed that it can operate. They need to give this detail and they need to state the true throughput of their system applying the weights.

Authors' response

In all the RC based systems, inference processing speed is governed by two different parts. The first is the processing speed of the RC core (physical nodes) and the second is the processing speed of the output layer applying the trained weights to the RC's outputs. Most physical RC systems are limited by the bandwidth of their nodes, therefore in this part of the system (RC core), our proposition relies on passive components that can offer ps time scales (THz bandwidth) which is clearly shown in the paragraph related to dispersion equalization in high baud rate transmission (100 Gbaud). In the vast majority of physical RCs, the outputs are processed in the digital domain. This part consists of detecting/sampling and applying weights at the RC outputs. Therefore, processing speed is only limited by the electro-optic conversions at the receiver. Assuming state of the art digital electronics, processing speed at this part can reach up to 200 Gsa/sec. In this context, a highly parallelized pipelined configuration can permit the operation with marginal latency even at very high baud rates (exceeding 100 Gbaud). This is an established approach in

typical equalizers (FFE, DFE) already used in high-speed optical communication systems [3]. Alternatively, there are few approaches that employ an analog readout layer, which is discussed in the supplementary document. In this case, the weights are applied during inference through properly biasing phase shifters and thus processing speed is performed at the speed of light. Summarizing, the proposed RC core imposes no processing speed limits, whereas in the case of a digital output layer, parallelization can offer processing speed that can match the RC core. The only existing bottleneck is digitization.

We have modified the introduction with the following part: *"Processing speed is capped only by the bandwidth of the photodiodes and the analog to digital conversion. A ROSS-NN can be incorporated either as an RNN or spatial RC architecture, providing direct coherent processing without costly electro-optic conversions, speed processing penalty, high-speed pseudo-random generators for mask realization [13] and most importantly, with marginal power consumption. In the RC case, the application of weights is implemented at a digital linear output layer. Thus, through parallelization, weighting of the RC outputs (multiplication and accumulation) can be performed with a speed matching the digitization process."*

Reviewer's comment 4

4) They do not explain <why> their system is equivalent to a reservoir computer. The filtering is equivalent, in the time domain, to performing a convolution of the filter function (in the time domain) with the incoming signal (in the time domain). There are several works that describe how convolutions, via a Volterra series expansion, can be a way to represent any function. The authors should consult this older reference: Boyd et al, IEEE Trans. Circuits Sys. CAS-32, 1150 (1985) and more recent works such as Gonon et al., IEEE Trans. Neural Netw. Learn. Syst. 31, 100 (2020), which should help them explain why their method works.

Authors' response

We would like to thank the reviewer for this fruitful comment and the really helpful references indicated. In the original manuscript, we have already claimed why our approach is equivalent to RC computer. We have written that "*an intuitive way to understand RC operation, is as a nonlinear dynamical system that acts as a pre-filter on the input data, transforming it into a higher dimensional space*". Moreover, we have written that *"This filter-based neuromorphic assumption of nodes is inspired by the filter-and-fire model which treats each retinal ganglion cell as a linear filter followed by a nonlinear activation function"*

The reference of Gonon et al. suggested by the reviewer clearly states that very important features of causal and time-invariant filters like the fading memory property or universality are naturally inherited by reservoir systems. Our proposition constitutes of nodes that are causal and time-invariant optical filters that provide fading memory as shown in supplementary information and which have the echo state property, since the reservoir will asymptotically wash out any information from initial conditions due to the passive nature of the recurrent filter (optical feedback below 1) which precludes instabilities in the dynamical behavior. A similar text has been added in page 5 of the revised manuscript so as to better clarify the relevance of ROSS-NN to RC.

Reviewer's comment 5

5) The idea of spectral filtering of signals to improve the performance of an RC has already been given in Carroll, Physica D 416, 132798 (2021). The authors need to put their work in the context of this previous work.

Authors' response

We believe that there is a pivotal difference between our proposal and what Carroll proposes. Our work introduces filters as building blocks of RNNs-RCs and not as post-processing units after RC in the readout. On the contrary, Carroll describes the use of FIR filters after the RC, acting in practice as an expanded linear readout layer. In fact, this is not a novel concept, as many proposed physical reservoir computers take advantage of expanded readout layers, especially in telecom applications where the similarity of the extended linear regression with a fractionally spaced Feed Forward Equalizer (FFE) is obvious. Indeed, we use exactly a FFE after the reception and digitization of the filter outputs, as we (see ref. 20 of the original manuscript) and others [8] also did in older publications, in order to provide an amount of external memory that a few physical-node RC system cannot reach. So, Caroll proposes an external memory increase through a digital FIR filter implemented in the digital domain (output layer), something that has been used by other researchers (and by us in this work with the use of FFE) and it is not relevant to what we propose as a novel contribution to the field.

We have added a reference to Carroll's work in order to further support the choice of using a simple FFE at the output layer in order to extract 100 Gbaud signals.

Reviewer's comment 6

Overall, I see very little improvement in our understanding of machine learning in this manuscript - they appear to be repackaging what is already known in the literature and they don't give a compelling explanation as to why their method works. So the main possible reason for publication is that they truly have a high-throughput system. But they do not present compelling evidence that they have done so unless they can demonstrate fast full-system throughput including applying the weights.

Authors' response

The main concept and significance of our paper is spectrally slicing a signal and manipulating it in a different manner per frequency band in recurrent processing. Based on ref. 22 of the manuscript, that proposes a simple spectral slicing of a signal and then machine learning processing, we propose the merging of recurrent processing and spectral slicing in the optical domain, inspired by the filter-and-fire model [ref. 21] of biological systems. For the first time, we explain what the merits and advantages of manipulating different spectral components are and how such a recurrent spectral processing is beneficial for a large category of problems, such as telecommunications or imaging. Especially in the power fading problem caused by chromatic dispersion in short-reach high baud rate systems, we illustrate the superiority of ROSS-NN over state of the art digital equalizers (MLSE, VNLE), which is a clear indication that spectral slicing enhances processing capabilities.

As for the throughput of the system, we have already answered in comment 3 and in the abstract of the revised manuscript that that the proposed neuromorphic accelerator that can easily scale to 200 GSa/sec

with state-of-the-art analog demultiplexing technologies [9], covering the needs of any ultra-fast application, in contrast to most RC works in the literature that deal with time-delayed systems.

Finally, a significant advantage, in comparison with other spatially distributed systems, is the utilization of spectral slicing that, in the general case, can reduce the bandwidth requirements of each node output. For example, an RC consisting of *N* spatial nodes that does not employ spectral slicing, as all the previous works in literature, needs *N* separate receivers of bandwidth *BW* where *BW* the bandwidth of the signal to be processed. On the contrary, our proposition requires a bandwidth of BW/N per node at the output layer as a result of spectrum slicing process. With recurrent spectral slicing, a simple RC with off-the-shelf electronics (20 GHz bandwidth) can easily handle and process multi-THz optical signals.

We have modified specific parts in the introduction and in the discussion in order to better highlight the unique properties of ROSS-NN that solidify its novelty.

Reviewer's comment 7

The error rates on the tasks are fairly high. The tasks are simple enough that a linear classifier can do a reasonable job. To show that their method does better than a linear classifier, which is a minimum baseline, the authors need to compare their error rates to a linear classifier.

Authors' response

We have tried to better clarify the difficulty of the tasks in the revised manuscript.

This work applies the same concept in two different problems, addressing two similar architectures. In the first case, a benchmark task (NARMA-10) is addressed with 4 to 30 nodes separated in 2 to 6 banks. The NMSE results acquired for this number of nodes are much better than those provided by a linear classifier (NMSE ~ 0.16 based on ref. 13 of the manuscript and according to our findings) and comparable to the state-of-the-art of RC implementations. In the revised manuscript, a comparison is carried out over three state of the art numerical works in the literature at the same node count proving this claim.

In the second, extremely difficult, task of chromatic dispersion compensation in ultra-fast optical communication systems, a two-node accelerator equipped with a FFE as the output layer, performs several orders of magnitude better than the linear algorithm/classifier (FFE) in terms of BER and about one order of magnitude better than the heaviest and optimal digital equalizer (MLSE). An extensive comparison is carried out in the paragraph entitled as *"Few-node ROSS-NN as a photonic hardware accelerator in 100 Gbaud and beyond optical communication systems"*, while figure 3b. clearly shows that the BER is below any standard method and in any published work, for the specific problem. Also in the self-coherent case, acceptable results are achieved in a very novel and unconventional way for complex modulation formats (16-QAM), as the system alleviates the need for coherent reception and retrieves phase modulated data with simple photodetectors. Lastly in the Kerr-induced non-linear problem, the minimal two-node system outperforms the linear FFE offering 2 dB gain, which is in compliance with very strong non-linear algorithms [10,11]. Therefore, we strongly believe that the proposed photonic accelerator offers both state-of-the-art results and ultrafast operation, addressing even the extremely demanding needs of next generation optical communications.

In conclusion, all comparative studies with respect to state-of-the-art systems and linear equalizers are clearly included in the revised manuscript, showcasing the very good performance of ROSS-NN approach. We have also included the Table I in order to better highlight this significant superiority of our study compared to similar photonic RC works in literature.

1. Mesaritakis, C., Sozos, K., Dermanis, D. & Bogris, A. Spatial photonic reservoir computing based on non-linear phase-to-amplitude conversion in micro-ring resonators. in *Optics InfoBase Conference Papers* (2021).
2. Coarer, F. D.-L. *et al.* All-Optical Reservoir Computing on a Photonic Chip Using Silicon-Based Ring Resonators. *IEEE Journal of Selected Topics in Quantum Electronics* **24**, 1–8 (2018).
3. Argyris, A., Bueno, J. & Fischer, I. PAM-4 Transmission at 1550 nm Using Photonic Reservoir Computing Post-Processing. *IEEE Access* **7**, 37017–37025 (2019).
4. Mesaritakis, C., Bogris, A., Kapsalis, A. & Syvridis, D. High-speed all-optical pattern recognition of dispersive Fourier images through a photonic reservoir computing subsystem. *Optics Letters* **40**, 3416 (2015).
5. van der Sande, G., Brunner, D. & Soriano, M. C. Advances in photonic reservoir computing. *Nanophotonics* **6**, 561–576 (2017).
6. Lugnan, A. *et al.* Photonic neuromorphic information processing and reservoir computing. *APL Photonics* **5**, 020901 (2020).
7. Freiberger, M. *et al.* Improving Time Series Recognition and Prediction With Networks and Ensembles of Passive Photonic Reservoirs. *IEEE Journal of Selected Topics in Quantum Electronics* **26**, 1–11 (2020).
8. Argyris, A., Bueno, J. & Fischer, I. Photonic machine learning implementation for signal recovery in optical communications. *Scientific Reports* **8**, (2018).
9. Thomas, P., Grozing, M. & Berroth, M. Analog Demultiplexer Operating at up to 200 GS/s Using Four Time Interleaved Switched Emitter Followers with a 50% Duty Cycle Clock. in *2021 IEEE BiCMOS and Compound Semiconductor Integrated Circuits and Technology Symposium, BCICTS 2021* (Institute of Electrical and Electronics Engineers Inc., 2021). doi:10.1109/BCICTS50416.2021.9682471.
10. Deligiannidis, S., Bogris, A., Mesaritakis, C. & Kopsinis, Y. Compensation of fiber nonlinearities in digital coherent systems leveraging long short-term memory neural networks. *Journal of Lightwave Technology* **38**, 5991–5999 (2020).
11. Sidelnikov, O., Redyuk, A., Sygletos, S., Fedoruk, M. & Turitsyn, S. Advanced Convolutional Neural Networks for Nonlinearity Mitigation in Long-Haul WDM Transmission Systems. *Journal of Lightwave Technology* **39**, 2397–2406 (2021).

REVIEWERS' COMMENTS:

Reviewer #1 (Remarks to the Author):

I would like to thenk the authors for the detailed discussion regarding all points raised in my original review. I can recommend the new version of the manuscript for publication.

Reviewer #2 (Remarks to the Author):

I am satisfied that the authors have taken my comments into account in the latest version of the manuscript and I believe the manuscript is appropriate to publish, provided that the other two referees also agree that their concerns have been addressed.

Reviewer #3 (Remarks to the Author):

I think the authors have done a good job of addressing the concerns of all of the referees. They have greatly improved the work. I can now recommend it for acceptance in its current form.
